# MoS_2_ Nanoplatelets on Hybrid Core-Shell (HyCoS) AuPd NPs for Hybrid SERS Platform for Detection of R6G

**DOI:** 10.3390/nano13040769

**Published:** 2023-02-18

**Authors:** Shusen Lin, Rutuja Mandavkar, Shalmali Burse, Md Ahasan Habib, Tasmia Khalid, Mehedi Hasan Joni, Young-Uk Chung, Sundar Kunwar, Jihoon Lee

**Affiliations:** 1 Department of Electronic Engineering, College of Electronics and Information, Kwangwoon University, Nowon-gu, Seoul 01897, Republic of Korea; 2 Center for Integrated Nanotechnologies (CINT), Los Alamos National Laboratory, Los Alamos, NM 87545, USA

**Keywords:** surface-enhanced Raman spectroscopy (SERS), HyCoS AuPd NPs, localized surface plasmon resonance (LSPR), MoS_2_ NPs, electromagnetic mechanism (EM), chemical mechanism (CM)

## Abstract

In this work, a novel hybrid SERS platform incorporating hybrid core-shell (HyCoS) AuPd nanoparticles (NPs) and MoS_2_ nanoplatelets has been successfully demonstrated for strong surface-enhanced Raman spectroscopy (SERS) enhancement of Rhodamine 6G (R6G). A significantly improved SERS signal of R6G is observed on the hybrid SERS platform by adapting both electromagnetic mechanism (EM) and chemical mechanism (CM) in a single platform. The EM enhancement originates from the unique plasmonic HyCoS AuPd NP template fabricated by the modified droplet epitaxy, which exhibits strong plasmon excitation of hotspots at the nanogaps of metallic NPs and abundant generation of electric fields by localized surface plasmon resonance (LSPR). Superior LSPR results from the coupling of distinctive AuPd core-shell NP and high-density background Au NPs. The CM enhancement is associated with the charge transfer from the MoS_2_ nanoplatelets to the R6G. The direct contact via mixing approach with optimal mixing ratio can effectively facilitate the charges transfer to the HOMO and LUMO of R6G, leading to the orders of Raman signal amplification. The enhancement factor (EF) for the proposed hybrid platform reaches ~10^10^ for R6G on the hybrid SERS platform.

## 1. Introduction

Surface-enhanced Raman spectroscopy (SERS) is a powerful optoelectronic analysis approach for the ultrasensitive detection of chemical and biomedical molecular species at extremely low levels of concentrations [1,2,3]. The SERS has offered fascinating opportunities for a wide range of applications such as optoelectronics, photocatalysis, gas sensors, energy harvesting, etc. [4,5,6]. Regarding SERS enhancement mechanisms, the electromagnetic mechanism (EM) has been widely adapted as a dominant mechanism for various configurations of metallic micro- and nano-structures [5,6,7,8]. The EM is induced by the photon-driven excitation of localized surface plasmon resonance (LSPR), enabling giant enhancement of local e-fields that are significantly stronger than the incident photon [9,10,11,12]. Plasmonic nanostructures demonstrated several orders of SERS amplifications and high resonance intensity by the substrate nanostructures and effective interaction with target molecules are the desirable characteristics [6,9,11]. Ag and Au are widely adapted plasmonic materials along with their superior LSPR resonance intensity, stability, and wide wavelength range in which most of Raman emission takes place [9,11,13]. Various Ag and Au-based plasmonic platforms have been widely explored for SERS enhancement such as Ag nanorods, porous AgAu NPs, Ag/GQD, Au NPs/MXene, and so forth [8,14,15,16,17,18,19,20]. The bimetallic hybrid NP configuration can provide extended options to alert the size- and shape-dependent LSPR [8,19,20]. At the same time, metallic Pd NP can be a promising plasmon-enhanced sensing platform due to superior NIR extinction and chemical stability [8,21]. However, Pd NPs are barely adapted as SERS substrate. Thus, developing bimetallic AuPd NPs by droplet epitaxy can be a novel route for SERS application. Meanwhile, the chemical mechanism (CM) enhancement has been widely adapted as a SERS mechanism for various configurations of semiconductor micro- and nano-structures [22,23,24,25,26]. The CM is related to the charge transfer from the semiconductor micro- and nano-structure substrates to probe molecules. Up to now, a variety of semiconductor materials, i.e., TiO_2_, CdS, GQD, MoS_2_, ZnO, etc., have been demonstrated as promising SERS substrates with the CM enhancement [23,24,25,26]. Among them, MoS_2_, a transition metal dichalcogenide (TMD) semiconductor with good chemical stability and activity, has gained extensive research attention in chemical catalysts, energy conversion, biochemical sensing, and SERS [27,28]. MoS_2_ nanostructures can offer superior molecular interactions due to the covalent Mo and S bonds with numerous absorption edges and thus can contribute to the high charge transfer for strong SERS enhancement [27,29]. For example, Quan et al. demonstrated an ultrasensitive SERS detection of bilirubin by MoS_2_@ZnO@Ag template where the SERS enhancement by the charge transfer between MoS_2_ and bilirubin can be effectively achieved by the CM enhancement [30]. In short, the development of bimetallic AuPd NPs can be a viable approach to induce a strong EM-based SERS enhancement and strong CM enhancement can be exploited by MoS_2_ nanostructures to increase an originally weak Raman signal. To this end, the design of a SERS platform that involves both HyCoS AuPd NPs and MoS_2_ nanoplatelets in a single SERS substate can be a worthy approach to adapt both the CM and EM at the same time.

**Figure 1:**
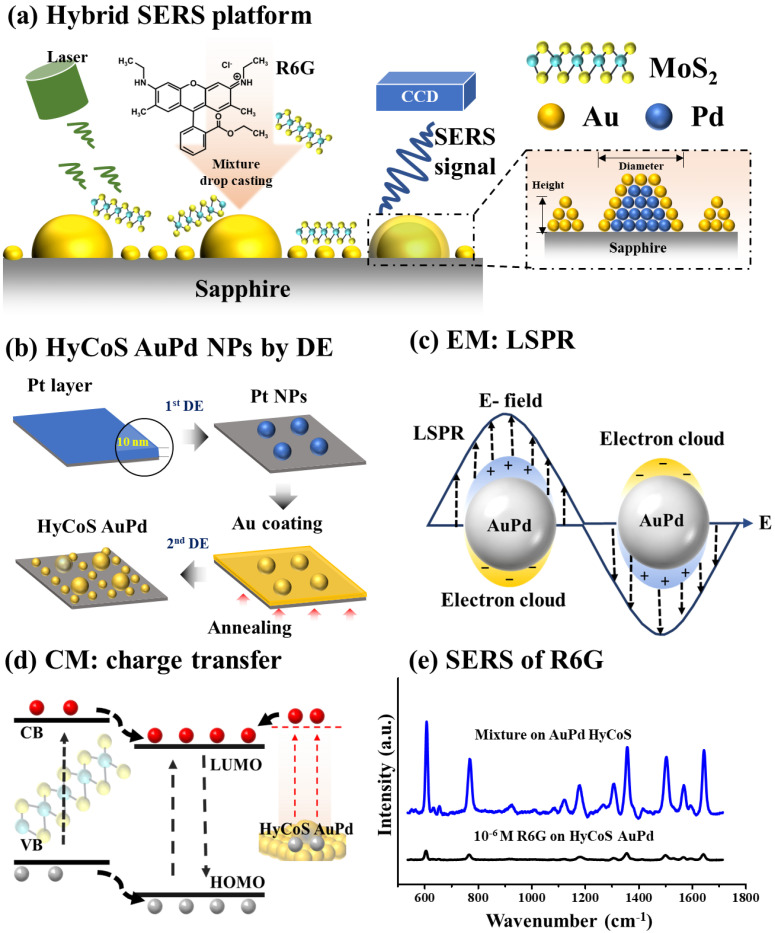
(**a**) Hybrid surface-enhanced Raman spectroscopy (SERS) platform by adapting electromagnetic mechanism (EM) and chemical mechanism (CM) simultaneously through the combination of hybrid core shell (HyCoS) AuPd nanoparticles (NPs) and MoS_2_ nanoplatelets. (**b**) Fabrication of AuPd HyCoS NPs by droplet epitaxy (DE). (**c**) Schematic of electromagnetic mechanism (EM) by localized surface plasmon resonance (LSPR) from the AuPd HyCoS NPs. (**d**) Schematic of charge transfer. (**e**) SERS enhancement of Rhodamine 6G (R6G) by hybrid SERS platform.

In this work, a hybrid SERS platform combining hybrid core-shell (HyCoS) AuPd NPs, and MoS_2_ nanoplatelets has been successfully developed for the Raman signal enhancement of R6G via the synergetic effect of EM and CM together as seen in Figure 1a. Unique plasmonic HyCoS AuPd NPs with core-shell AuPd NPs and high-density background Au NPs are fabricated by a modified droplet epitaxy as illustrated in Figure 1b. The EM enhancement results from the intense excitation of LSPR on the plasmonic HyCoS AuPd NPs as seen in Figure 1c. The LSPR properties of various HyCoS AuPd NPs have been systematically investigated by using surface morphological, optical characterization, and FDTD simulation. The CM enhancement is achieved by the charge transfer from MoS_2_ nanoplatelets to the HOMO and LUMO levels of R6G as seen in Figure 1d. Significant SERS enhancement of R6G is demonstrated by adapting both CM and EM through the mixing approach reaching enhancement factor (EF) of ~10^10^ as compared with the bare HyCoS AuPd NPs in Figure 1e. 

## 2. Fabrication of Hybrid Core-Shell (HyCoS) AuPd NPs

**Figure 2:**
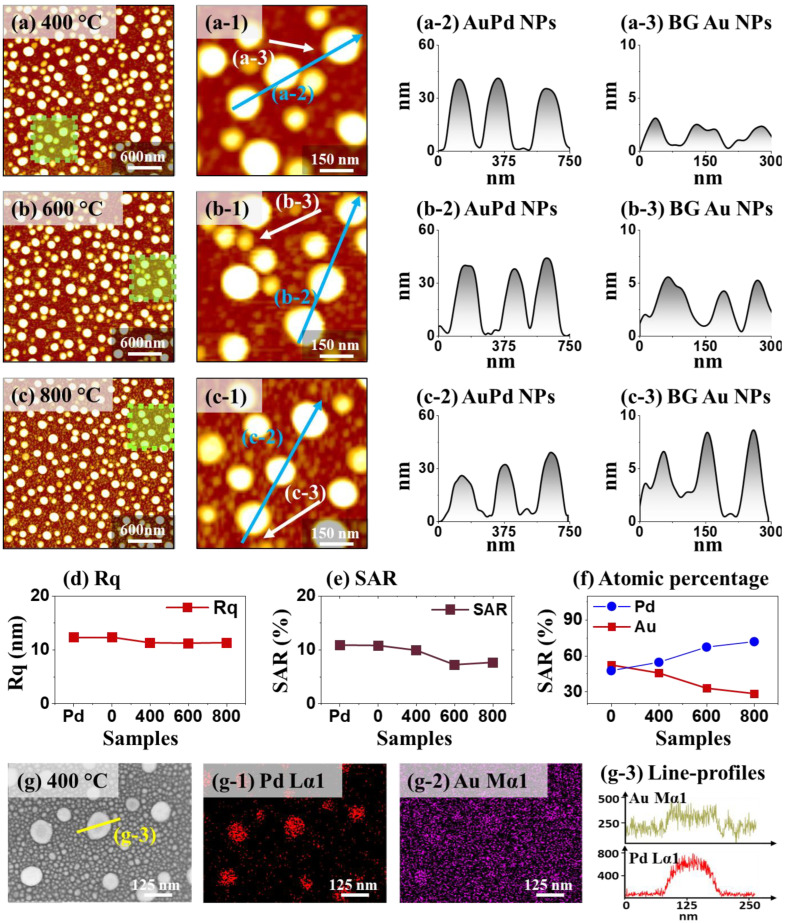
Hybrid core-shell (HyCoS) AuPd NPs by 3 nm Au coating and annealing at 400, 600, and 800 °C by modified droplet epitaxy (DE). Pd NP template was fabricated with 10 nm Pt film by 1st stage DE. (**a**–**c**) AFM top-views of AuPd NPs by 2nd stage DE. (**a-1**–**c-1**) Enlarged AFM top-views. (**a-2**–**c-2**) Line-profiles of AuPd NPs (blue arrows). (**a-3**–**c-3**) Line-profiles of background (BG) Au NPs. (**d**–**f**) Plots of Rq, SAR, and atomic percentages of AuPd HyCoS NPs. (**g**) SEM image of HyCoS AuPd NPs at 400 °C (**g-1-g**-2) EDS phase maps of Pd and Au. (**g-3**) Corresponding line-profiles as indicated in (**g**).

Figure 2 shows the surface morphology evolution of hybrid core-shell (HyCoS) AuPd nanoparticles (NPs) by 3 nm Au coating on Pd NP template at different annealing temperatures. In the first step, the Pd NP template was developed by the modified droplet epitaxy (DE) through solid-state dewetting (SSD) process via annealing 10 nm Pd film at 800 °C for 450 s. To achieve well-isolated metallic NPs, Tamman temperature (*T*_Tammam_) is generally selected for the SSD, at which the adatoms can become active for surface diffusion to find the crystal lattice at an appropriate annealing condition [12]. The *T*_Tammam_ is about half of the absolute metal melting point (*T*_melting_). The T_melting Pd_ is 1555 °C [31] and therefore, the optimal dewetting temperature can be estimated to be ~800 °C, considering the heat loss in the vacuum system. Followed by the annealing, Pd adatoms can diffuse and the isolated Pd NP template can be successfully developed through the formation of metallic droplets by the DE as seen in Figure S2, induced by atomic diffusion by the surface energy minimization [8,16,32]. The average height and diameter of Pd NPs were ~35 and 135 nm. Subsequently, 3 nm Au film was sputtered on the Pd NP template to fabricate the HyCoS AuPd NPs by the 2nd stage DE. More detailed morphological analysis on 3 nm Au-coated Pd NP template before DE is provided in Figure S3, where no apparent change in shape and slight increase in NP height were observed. During the 2nd DE process, the coated Au adatoms on Pd NPs can go through diffusion process toward the high chemical potential sites of Pd NPs activated by thermal energy, indicating the formation of core-shell structure [33]. At the same time, the thin Au film possessing nanometric fluctuations can easily form stable NPs itself with smaller size in the background via inward diffusion and surface energy minimization, leading to a high density of background (BG) Au NPs [7,16]. The high-density BG NPs along with the core-shell AuPd NPs in the primary Pd NP sites are indicated as the hybrid core-shell (HyCoS) AuPd NPs. 

**Figure 3:**
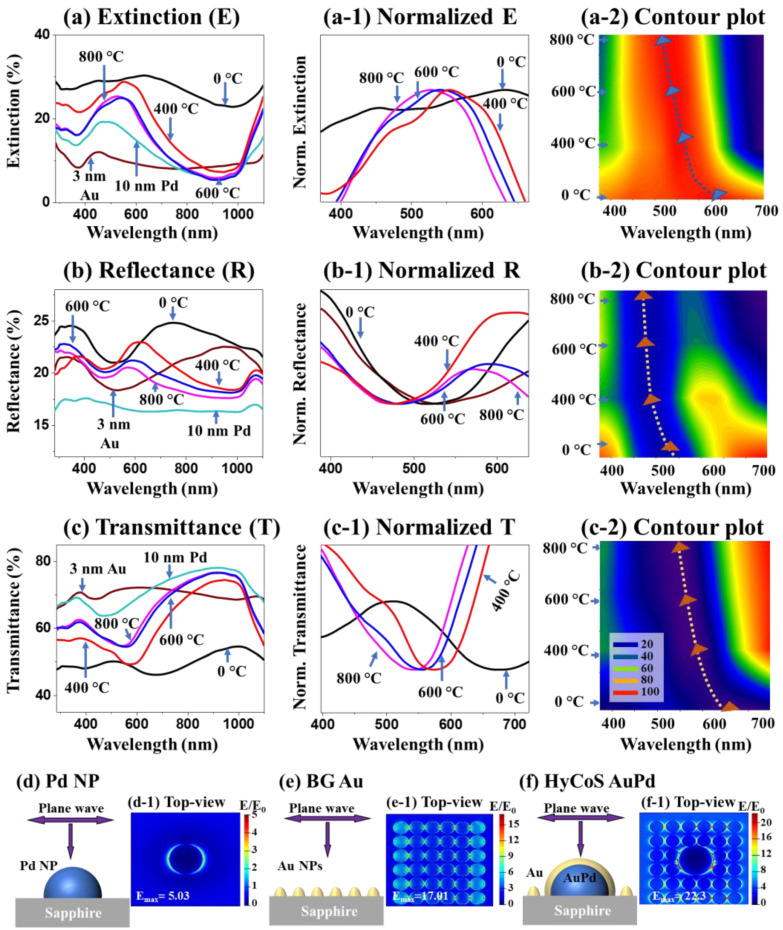
Optical properties of AuPd HyCoS NPs with 3 nm Au coating. (**a**–**c**) Extinction (E), reflectance (R) and transmittance (T) plots in the range of 300–1100 nm wavelength region. (**a-1**–**c-1**) Normalized E, R, and T plots. (**a-2**–**c-2**) Corresponding contour plots. (**d**–**f**) FDTD schematic representation for typical Pd, background (BG) Au and HyCoS AuPd NPs. (**d-1**–**f-1**) E-field distribution profile top-views.

The surface morphology evolution of HyCoS AuPd NPs at different annealing temperatures is shown in Figure 2a–c. The Pd NPs were stably retained regardless of the DE temperature. The zoom-in images and corresponding line-profiles are shown in Figure 2(a-1–c-1) and Figure 2(a-2–c-2) with slightly increased NP diameter. Gradually decreased height of core-shell NP can be due to the enhanced diffusion of Au atoms, which is the characteristic of thin-film dewetting by the DE [7,33]. Nevertheless, as seen in Figure 2(a-3–c-3), the secondary BG Au NPs showed increased size at higher annealing temperatures as the formed Au NPs have a tendency to absorb the adjacent adatoms and can grow further [12]. AFM side-views and histograms of height and diameter of this set can be found in Figure S4. It was clearly observed that the average height and diameter of core-shell AuPd NPs gradually decreased along with the increased SSD temperature. The overall transition in the morphology of NPs was evaluated by RMS surface roughness (Rq) and surface area ratio (SAR) as seen in Figure 2d–e [7]. The Rq represents the root mean squared height and can be calculated by the following equation: $Rq=\sqrt{\left(\frac{1}{n}\overset{n}{\underset{1}{\sum }}H_{n}^{2}\right)}$, where the H_n_ is the height profile at each pixel [7]. The SAR represents the ratio of 3D surface area (A_s_) to the geometric area (A_g_) by the relation, SAR = $\frac{\mathrm{Ag}\;-\;\mathrm{As}\;}{\mathrm{Ag}}$ × 100 % [33]. Here, the dominant effect can be related to the size change of core-shell NPs since it is more significant over the BG Au NPs [12] and thus the Rq and SAR both demonstrated decreasing trends along with the increased annealing temperature. The core-shell NP size appeared to decrease since the BG NPs were growing. The atomic percentage of this set is summarized in Figure 2f and the corresponding EDS spectra are provided in Figure S5. The increased Pd % can be explained by the reduction of Au % by the sublimation at high annealing temperature and vapor pressure [34]. 

Figure 2g shows the SEM image of HyCoS AuPd NPs at 400 °C, exhibiting the primary core-shell AuPd NPs surrounded by the high-density of background Au NPs. Figure 2(g-1–g-2) show the EDS elemental phase-maps of Pd Lα1 and Au Mα1, which demonstrated a good match with the corresponding SEM image. The Pd atoms were well retained in the primary NP sites whereas Au atoms were present in both the primary and background NP sites. The EDS elemental line-profiles are shown in Figure 2(g-3), where the intensity of Au was found everywhere with higher counts in the primary NP sites while the Pd counts were only found at the primary NP sites. This indicated the preferential diffusion of Au atoms towards the Pd NPs that possess the lower surface energy or high chemical potential as discussed as well as the formation of secondary background Au NPs. The phase maps of sapphire substrate (Al_2_O_3_) are shown in Figure S6 with Al Kα1 and O Kα1. The diameter distribution histogram of background Au NPs can be found in Figure S6c and the average diameter was estimated to be ~22 nm. Similarly, the HyCoS AuPd NPs fabricated with 5 nm Au coating were studied as shown in Figures S7–S10. The morphology evolution trend was like in the 3-nm Au-coating set. The size of BG Au NPs was larger than the 3-nm Au-coating set due to more available Au adatoms. The morphology evolution by the deposition of thicker Au film such as 10 nm was also investigated as seen in Figures S11–S14, which exhibited the complete alloyed diffusion process of Au and Pd adatoms without the formation of background Au NPs and interconnected and irregular AuPd NP clusters were fabricated in micron scale. Different diffusion behavior of evolution trend in the 2nd stage DE can be explained by the promoted intermixing process at the Pd/Au interface with the increased Au adatom amount and miscibility between Au and Pd atoms [12,33]. With a much increased amount of Au adatoms available with 10 Au deposition, the intermixing at the Pd/Au interface can be significantly enhanced. Due to the miscibility of Au and Pd atoms, the whole Pd NP matrix can be intermixed, destroying the Pd NPs, and overall mixed- or alloyed-phase diffusion can occur. As a result, much larger well-alloyed AuPd NP clusters can be formed as confirmed by the EDS maps in Figure S13 without the BG Au NP formation. Overall, the resulting nanostructures can be efficiently controlled by changing the thickness of the deposited Au layer on the Pd NP template and growth conditions in the second DE step.

### Optical Characterization of HyCoS AuPd NPs

Figure 3 shows the optical properties of HyCoS AuPd NPs with 3 nm Au coating. Here, the reflectance (R) and transmittance (T) spectra were measured within UV–Vis–NIR (300–1100 nm) and extinction spectra were extracted using the following relation: E% = 100% − (R% + T%) [35]. As seen in Figure 3a, the 3 nm Au film on sapphire (3 nm Au) showed an extinction peak at ~450 nm due to the localized surface plasmon resonance (LSPR) induced by the nanoscale film. The Pd NPs (10 nm Pd) demonstrated a broad visible peak with the peak center located at ~490 nm, which can be related to the dipolar resonance (QR) and quadrupolar resonance (QR) modes due to excitation of LSPR [33,36]. The 0 °C sample (3-nm Au-coated on Pd NPs before annealing) demonstrated a broader resonance peak extending into the visible region, likely resulting from the Au nano-grains and also defect formation in the Au film due to the deposition at an ambient temperature [7,33]. Along with the increased annealing temperature, the extinction spectra exhibited a blue shift from 400 to 800 °C as clearly seen by the normalized extinction plot and contour plot in Figure 3(a-1–a-2) [12,33]. The decreased LSPR peak intensity was slightly decreased at high dewetting temperatures [16]. The corresponding reflectance and transmittance spectra were shown in Figure 3b–c, which generally matched well with the extinction plots. The dips in the R-T plots correspond to the peaks in the E plot. Reflectance dips were observed in lower wavelengths, resulting from the backscattering of HyCoS NPs [37]. The R and T spectra both followed the blueshift trend as clearly seen in Figure 2(b-2–c-2) and Figure 3(b-1–c-1). The normalized E-R-T plots clearly demonstrated matched evolution trends of dips, peaks and bandwidth. The optical properties of AuPd NPs with 5 and 10 nm Au coating can be found in Figures S10 and S15. In short, the HyCoS AuPd NPs formed at 400 °C demonstrated the strongest extinction properties, which can indicate the strongest LSPR properties.

The LSPR properties of various metallic nanostructures are examined by utilizing finite-difference time-domain (FDTD) simulation. Here, the total-field scattered-field source was adapted as a light source and the incident wave was perpendicular to the metallic NPs to generate electromagnetic hotspots. More details on the simulation settings can be found in the Supplementary Section S1.5 and additional simulations with top- and side-views can be seen Figure S16. The schematics of pure Pd, background (BG) Au, and HyCoS AuPd NPs are shown in Figure 3d–f and the corresponding top-views of e-field distribution are displayed in Figure 3(d-1–f-1). From the pure Pd NPs by the DE in Figure 3d, the maximum intensity of electromagnetic field was observed at the boundary of NP [33]. The pure Pd NP demonstrated the maximum local e-field intensity (MLEI/E_max_) of 5.03 in Figure 3(d-1). This was increased to 7.01 for Au NPs in Figure S16(b-1). To probe the background NP effect, the high density of Au NPs is separately simulated in Figure 3e. The high-density small Au NPs exhibited the MLEI of 17.01 in Figure 3(e-1), which was ~2.4 times over the pure Au NPs, indicating small high-density NPs can demonstrate much higher local e-field intensity with largely increased hotspot density, which can be much beneficial for the SERS enhancement [33]. Meanwhile, the alloy AuPd NPs demonstrated the MLEI of 11.3 in Figure S16d. Finally, the HyCoS AuPd NPs demonstrated a significantly increased MLEI value of 22.3 in Figure 3(f-1), which was 3.7 times higher than the pure Pd NP. The large MLEI enhancement can be due to the strongly localized e-field in the nanogaps via EM coupling between the BG Au NPs [38]. Moreover, the core-shell configuration demonstrated increased LSPR as the maximum intensity of e-field distribution was found at the edge of AuPd core-shell NPs as seen from the side-view in Figure S16(e-1). The HyCoS AuPd NPs demonstrated the highest LSPR excitations due to the unique configuration of core-shell NPs and high-density BG NPs, which is suitable for the plasmonic SERS substrate. 

## 3. SERS Analyses of Rhodamine 6G on Various Substrates

**Figure 4:**
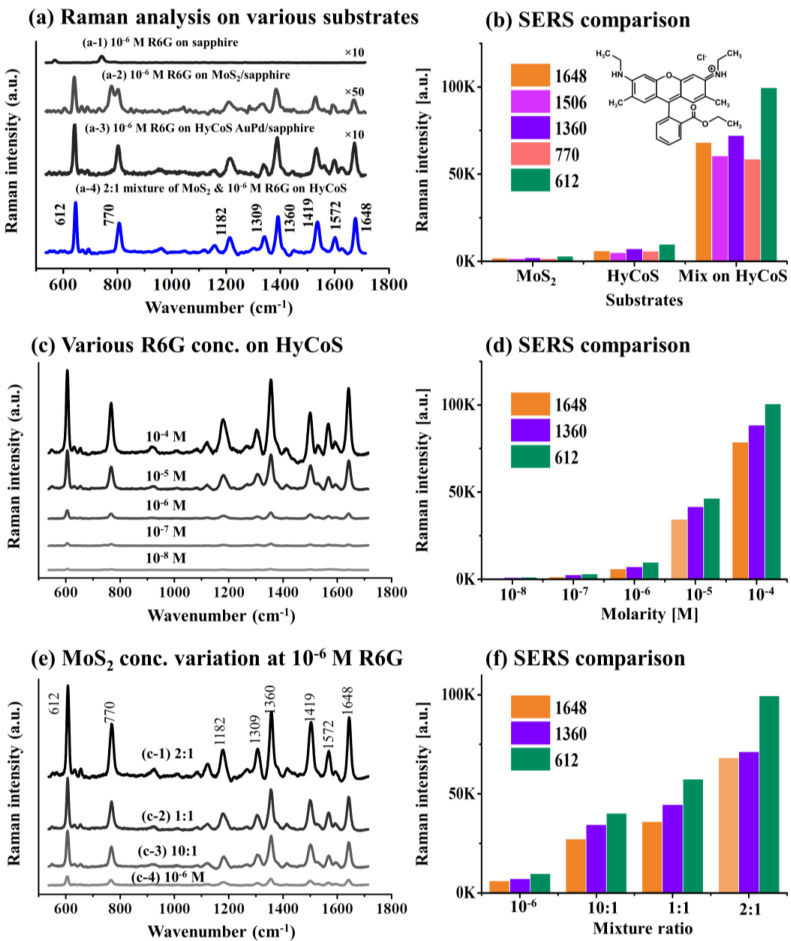
(**a**) Raman and SERS spectra of Rhodamine 6G (R6G) on various substrates as labeled. (**b**) Intensity counts of characteristic peaks. (**c**) SERS spectra by different concentrations of R6G on AuPd HyCoS NP substrate. (**d**) Intensity comparison of characteristic peaks. (**e**) SERS spectra of various mixture ratios of MoS_2_ nanoplatelets and R6G. (**f**) SERS enhancement comparison.

Figure 4 shows Raman and SERS analyses of Rhodamine 6G (R6G) on various substrates. According to the experimental and FDTD results, the HyCoS AuPd NP template with 3 nm Au coating annealed at 400 °C demonstrated the best LSPR properties and hence was selected as a plasmonic NP template for SERS application. Figure 4a shows the SERS signals of R6G on bare sapphire, MoS_2_ nanoplatelets, HyCoS AuPd NP substrates and the SERS enhancement is compared in Figure 4b. Specific values of this set are provided in Table S1. Firstly, the 20 µL 10^−6^ M R6G solution was drop-casted on sapphire in Figure 4(a-1), where no Raman signal of R6G was observed as sapphire is not a SERS substrate [8]. The peaks located at 573 and 746 cm^−1^ can be related to the E_g_ vibrational modes of sapphire [7]. Then, 20 µL 10^−6^ M R6G was applied on the MoS_2_ nanoplatelets/sapphire in Figure 4(a-2). MoS_2_ nanoplatelet powder was dissolved in ethanol to make 0.25 mg/mL. A series of intense SERS peaks of R6G such as 612, 770, 1360, and 1419 cm^−1^ were observed along with additional peaks. The R6G application on MoS_2_ nanoplatelets induced the SERS of R6G by the chemical mechanism (CM) enhancement through the charge transfer [39,40]. Additional peaks might have been induced by the edge defects from the MoS_2_ nanoplatelets [39]. The SERS enhancement factor of R6G by MoS_2_ nanoplatelet was ~10^8^ as calculated in Table S2. Then, the 10^−6^ M R6G was applied on the HyCoS AuPd NPs in Figure 4(a-3). Again, a strong long-range SERS enhancement was observed with several characteristic peaks of R6G. The SERS enhancement factor of R6G by the HyCoS AuPd NPs was ~10^9^ as calculated in Table S2, indicating the strong SERS effect by the HyCoS plasmonic template through the electromagnetic mechanism (EM) enhancement by the strong hotspots and electromagnetic fields through LSPR [7,41]. Subsequently, the volume ratio of 2:1 mixture solution of MoS_2_ and 10^−6^ M R6G in total 20 µL was applied on the plasmonic HyCoS AuPd NP template in Figure 4(a-4). Surprisingly, a significant SERS enhancement was achieved by the mixture application of MoS_2_ and R6G on the HyCoS NP. In this case, the SERS enhancement can be related to the concurrent enhancement by both chemical mechanism (CM) and electromagnetic mechanism (EM) [8,42,43]. The SERS intensity counts of characteristic peaks are shown in Figure 4b and the mixing approach demonstrated a much-enhanced SERS response.

To probe the SERS behavior of the plasmonic HyCoS AuPd NP template, different molarity of R6G ranging from 10^−4^ M to 10^−8^ were applied as seen in Figure 4c. Along with the increased molarity, the SERS peak intensity was gradually intensified as well summarized in Figure 4d. Specific values of this set are provided in Table S3. Gradual SERS enhancement can be due to the gradually increased molecular concentration by the effective interaction between the target molecules and high density hotspots induced by LSPR of HyCoS AuPd NPs [44]. The hot carriers or electrons excited by the strong LSPR and numerous hot spots on the HyCoS AuPd NPs can effectively enhance the carriers in the highest occupied molecular orbital (HOMO) of R6G molecules. Then, the electron transition to the lowest unoccupied molecular orbital (LUMO) energy levels of R6G molecules can be significantly enhanced with the numerous hot carrier transitions, resulting in significantly improved SERS signals. Obviously, the SERS counts can be increased with the increased molar concentration of R6G, which shows that the HyCoS AuPd NP is an effective SERS substrate. The prepared plasmonic template can sensitively detect the R6G molecules down to 10^−8^ M. Generally, the SERS enhancement by the metallic NP substrate is known as electromagnetic mechanism (EM) enhancement [7,41]. 

Figure 4e shows the ratio variation of MoS_2_ nanoplatelet with 10^−6^ M R6G on the HyCoS AuPd NPs. The total amount of measurement was fixed at 20 µL and the MoS_2_ nanoplatelet was in 0.25 mg/mL. AFM, EDS and Raman characterizations of MoS_2_ nanoplates on bare sapphire is provided in Figure S17. From the SERS signal of 10^−6^ M R6G on the HyCoS AuPd NPs in Figure 4(e-4), the SERS signals were significantly increased along with the mixture application of MoS_2_ and R6G in Figure 4(e-1–e-3). The SERS enhancement is summarized in Figure 4f and specific values of this set are provided in Table S4. The 2:1 ratio demonstrated the maximum SERS intensity compared to the other two mixture ratios and the signals were enhanced nearly about 10 times. With the lower MoS_2_ amount (10:1), the SERS enhancement was slightly lower likely due to the less charge transfer by the lower amount. With higher MoS_2_ amount (1:1), too high-density MoS_2_ nanoplatelet can absorb the SERS from the R6G molecules. Along with the mixture application, the carriers from the MoS_2_ nanoplatelets can be transferred to the HOMO of R6G molecules. Then, the increased carrier concentration can increase the transition from the HOMO to the LUMO and carrier excitation can also be efficiently improved within the R6G. This overall can result in significantly increased SERS enhancement. This type of SERS enhancement by semiconductor materials is known as chemical mechanism (CM) enhancement [8,42,43]. In the mixture approach, both the EM and CM were utilized along with the mixture of MoS_2_ nanoplatelet and R6G probe molecules on the HyCoS AuPd NPs. In addition, the 2:1 mixture application of MoS_2_ nanoplatelets and R6G on the HyCoS AuPd NPs for lower molar concentrations of R6G in Figure S18. Specific values of this set are provided in Table S5. With 10^−7^ M R6G, the enhancement was reduced to ~3 times in Figure S18a,(a-1). At 10^−8^ M, the output exhibited a similar intensity as seen in Figure S18b,(b-1). For the lower concentrations of R6G, the enhancement was lower and at 10^−8^ M, the CM enhancement was no longer effective.

### SERS Enhancement Factor (EF) and Enhancement Mechanism

**Figure 5:**
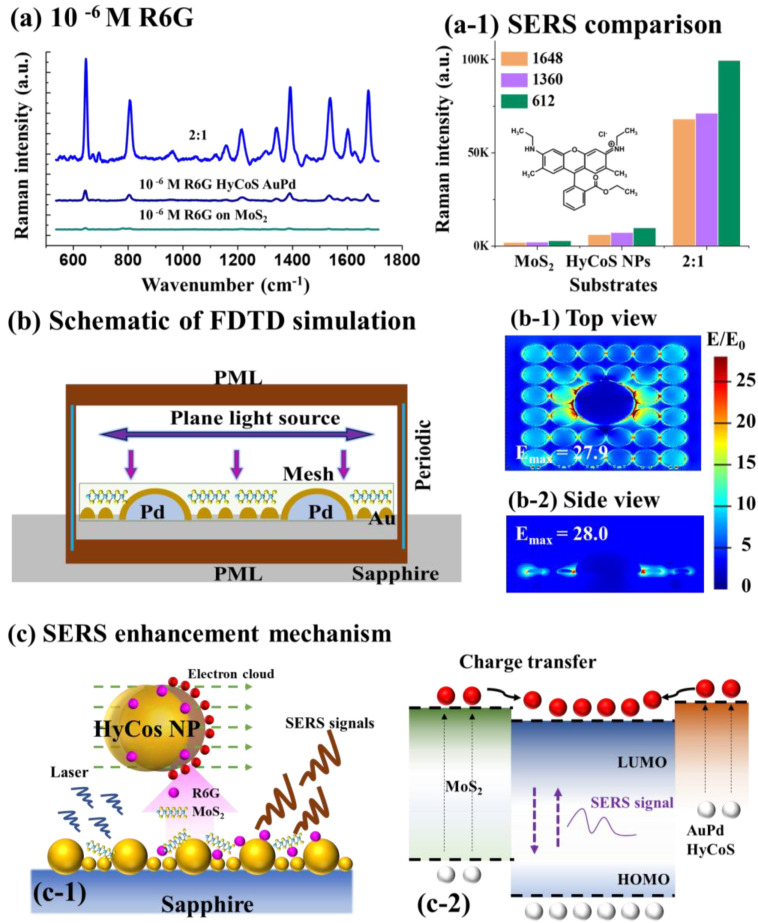
(**a**) Overall SERS intensity comparison of 10^−6^ M R6G on various substates: i.e., MoS_2_, AuPd HyCoS NPs and 2:1 mixture on AuPd HyCoS NPs. (**a-1**) Intensity counts of characteristic peaks. (**b**) Schematic of FDTD simulation of hybrid SERS platform. (**b-1**–**b-2**) Top- and side-views of e-field distributions. (**c**) SERS enhancement mechanism. (**c-1**) Schematic of R6G Raman signal enhancement by the hybrid SERS platform. Schematic of Raman signal enhancement by electromagnetic mechanism (EM) and chemical mechanism (CM). (**c-2**) Schematic of charge contributions by the MoS_2_ nanoplatelets and plasmonic HyCoS AuPd NPs.

Figure 5a shows the summarized comparison of 10^−6^ M R6G SERS signals on different substrates. The SERS enhancement was increased in the order of MoS_2_ nanoplatelets < HyCoS AuPd NPs < 2:1 mixture application of MoS_2_ nanoplatelets and R6G on the HyCoS AuPd NPs as summarized in Figure 5(a-1). The MoS_2_ nanoplatelets by the CM and HyCoS AuPd NPs by the EM can individually enhance the Raman signals of 10^−6^ M R6G. Based on that, the mixing approach demonstrated significantly improved SERS response, which was from the synergetic effect of the electromagnetic mechanism (EM) and chemical mechanism (CM) [45]. The optimal mixing ratio was found to be 2:1 as the appropriate amount of MoS_2_ nanoplatelets can guarantee sufficient adsorption sites for the charge transfer for the maximum enhancement of Raman signal as discussed [27]. The corresponding bar graph in Figure 5(c-1) clearly demonstrates the sharp increase by adapting the mixing approach. Further, the SERS enhancement factor (EF) of R6G was calculated for quantitative analysis for various SERS platforms, which was based on the SERS of R6G and normal Raman signal of R6G, using the following equation: $EF=\frac{I_{SERS}N_{Raman}}{N_{SERS}I_{Raman}}$, where I_SERS_ and I_Raman_ are SERS and Raman intensities of characteristic peaks [46,47]. The Raman spectrum of 10^−6^ M R6G on bare sapphire can be found in Figure S19 and more details on the EF calculation can be found in supplementary S.1.6. The SERS EF was calculated to be ~10^8^ for the case of 10^−6^ M R6G on the MoS_2_ nanoplatelets by the CM. The 612, 1360, 1648 cm^−1^ peaks showed the corresponding EF of 3.08 × 10^8^, 2.32 × 10^8^, and 3.12 × 10^8^ as summarized in Table S2. Then, the SERS EF was calculated to be ~10^9^ for the case of 10^−6^ M R6G on the HyCoS AuPd NPs by the EM. The 612, 1360, and 1648 cm^−1^ peaks showed the corresponding EF of 31.56 × 10^9^, 7.79 × 10^9^, and 6.43 × 10^9^ in Table S2. Finally, the SERS EF was calculated to be ~10^10^ for the case of 10^−6^ M R6G on the 2:1 mixture, i.e., MoS_2_ nanoplatelets and R6G on the HyCoS AuPd NPs. The corresponding EF of 1.59 × 10^10^, 7.82 × 10^10^, and 7.19 × 10^10^ were calculated for the characteristic 612, 1360, and 1648 cm^−1^ peaks in Table S2. The mixing approach demonstrated two-order higher EF over the MoS_2_ nanoplatelets and one-order higher EF over the HyCoS AuPd NPs by taking both EM and CM on the on various characteristic peaks of R6G. In order to probe the detection ability of hybrid platform, the limit of detection (LOD) was calculated based on the following equation [48]: $LOD=\frac{3\sigma }{b}$, where the σ is residual standard deviation and b is the slope of regression line. The linear relationship in the range between 10**^−8^** to 10**^−6^** M with the 2:1 mixture MoS**_2_** and 10**^−6^** M R6G on AuPd HyCoS NPs is shown in Figure S20. The 1360 cm**^−1^** peak with the highest intensity was used for LOD calculation. σ was obtained based on 10-time blank readings and the LOD was calculated to be 6.68 × 10**^−10^** M.

The Raman signal enhancement mechanism for the hybrid SERS platform based on the MoS_2_ nanoplatelets and HyCoS AuPd NPs can be related to the electromagnetic mechanism (EM) and chemical mechanism (CM) as briefly discussed. To begin with, the FDTD simulation was carried out to probe the electromagnetic field distribution on the hybrid SERS platform, i.e., MoS_2_ nanoplatelets on the HyCoS AuPd NPs that included the core-shell AuPd NPs along with the high-density small background Au NPs. The FDTD schematic can be seen in Figure 5b, where the MoS_2_ nanoplatelets were placed on top of the metallic AuPd HyCoS NPs as in the experimental set-up. The plane-wave was vertically irradiated on the hybrid SERS platform for the local excitation of LSPR and the corresponding top and side-views of e-field distributions are shown in Figure 5(b-1–b-2). Here, the maximum local e-field intensity (MLEI/E_max_) of the hybrid SERS platform was ~28.0, which was largely improved from the 22.3 of plasmonic HyCoS AuPd NPs in Figure 3f. This indicates much-improved resonance e-field intensity along with the incorporation of MoS_2_ nanoplatelets. The schematic of R6G Raman signal enhancement by the hybrid SERS platform through the CM and EM is shown in Figure 5(c-1) and the schematic of charge contributions by the MoS_2_ nanoplatelets and plasmonic HyCoS AuPd NPs is shown in Figure 5(c-2). As seen by the FDTD simulation, the hybrid platform demonstrated the best maximum local e-field intensity by the plasmon resonance excitation due to the unique AuPd core-shell structure as well as high-density background Au NP coupling [16]. It is worth noting that the high-density background Au NPs can largely increase the hotspot density and interaction cross-section with the R6G molecules [16,33,49]. In this regard, a large number of hotspots and strong local electromagnetic fields can be generated by the laser irradiation, which can effectively boost the charger transfer from the AuPd HyCoS NPs to R6G molecules, suggesting a significantly amplified SERS response by the EM enhancement [44,50]. At the same time, the MoS_2_ nanoplatelets possess superior adsorption ability and a large number of electrons can be excited to the conduction band by laser irradiation. Moreover, the MoS_2_ nanoplatelets can offer sufficient adsorption edges for the probe molecules via the dipole–dipole coupling due to the presence of polar covalent bonds (Mo-S) [51,52]. The charge transfer can be effectively facilitated from the conduction band (CB) of MoS_2_ to the HOMO of R6G [50,53,54]. The charge transfer by both EM and CM can significantly amplify the Raman signals [54]. Consequently, under laser excitation, the hybrid platform of HyCoS AuPd NPs and MoS_2_ nanoplatelets by taking advantage of both CM and EM can remarkably enhance the original weak Raman signals of R6G.

## 4. Conclusions

In summary, the plasmonic HyCoS AuPd NPs have been successfully fabricated on a sapphire substrate (0001) by a two-step modified droplet epitaxy (DE) approach and the NP optimization process was based on thickness control of Au films and annealing temperature control in the second DE step. A unique HyCoS AuPd NP configuration, i.e., AuPd core-shell shell structure along with high-density background Au NPs, exhibited the highest resonance intensity in the optical measurement as well as FDTD simulation. The FDTD simulation was carried out to study the LSPR properties and it was found that a strong e-field occurred at the nanogaps and edges of metallic NPs. The SERS enhancement of R6G was observed both on MoS_2_ nanoplatelets as well as on the HyCoS AuPd NPs. Then, the mixture of MoS_2_ nanoplatelets and 10^−6^ M R6G demonstrated around two-order higher SERS signal as compared to the MoS_2_ nanoplatelet SERS and around one-order higher enhancement as compared to the HyCoS AuPd NP SERS. Various mixture ratios of MoS_2_ and R6G, i.e., 10:1, 1:1, and 2:1, were evaluated and 2:1 was found to be optimum. In the hybrid SERS platform based on the MoS_2_ nanoplatelets and HyCoS AuPd NPs, strong Raman signal enhancement was observed, which was based on the charge transfer by the MoS_2_ nanoplatelets and LSPR by the HyCoS AuPd NPs through both chemical mechanism (CM) and electromagnetic mechanism (EM) enhancement.

## Data Availability

No new data were created.

## Supplementary Materials

The following are available online at https://www.mdpi.com/article/10.3390/nano13040769/s1, the Supplementary Materials include additional morphological, elemental, optical, and Raman characterizations of various AuPd hybrid core-shell (HyCoS) nanoparticles (NPs) and alloy NPs including the AFM images, EDS spectra, optical spectra, and FDTD simulation, Figure S1: (a) Atomic Force Microscopy (AFM) side-view of bare sapphire (0001) degassed at 600 °C for 30 min. (b) Corresponding surface line-profile. (c) Transmittance spectra with ~ 84 % average transmittance. (d) Reflectance spectra with 13 % average reflectance; Figure S2: Fabrication of palladium (Pd) nanoparticles (NPs) by droplet epitaxy of 10 nm Pt film on sapphire (0001) at 800 °C for 450 s. (a) Large scale AFM top-view (3 × 3 µm2). (b) AFM side-view with corresponding line-profile. (c) Schematic representation of Pd NP formation by droplet epitaxy. (d) – (f) Extinction, transmittance and reflectance spectra of Pd template; Figure S3: Surface morphological characterizations of AuPd NPs with 3 nm Au coating before annealing. (a) – (a-1) AFM side-views (3 × 3 µm2) and corresponding line-profile. (b-1) – (b-2) AFM top-view, enlarged top-view and corresponding line-profile; Figure S4: (a) – (c) AFM side-views (3 × 3 µm2) of AuPd hybrid core-shell (HyCoS) NPs with 3 nm Au coating annealed at various temperatures. (a-1) – (c-1) Corresponding cross-sectional line-profiles. (a-2) – (d-2) Height distribution histograms of core-shell AuPd NPs. (a-3) – (d-3) Diameter distribution histograms of core-shell AuPd NPs; Figure S5: (a) – (d) EDS spectra of AuPd HyCoS NPs with 3 nm Au coating at different temperatures. (a-1) – (d-1) Zoom-in spectra of Au and Pd. (a-2) – (d-2) EDS elemental composition table for Au and Pd NPs with varying annealing temperature; Figure S6: (a) – (b) EDS phase maps of Al and O. Related to Figure 2(g). (c) Diameter distribution histogram of background Au NPs on bare sapphire; Figure S7: (a) – (d) AFM side-views (3 × 3 µm2) of AuPd HyCoS NPs with 5 nm Au coating annealed at various temperatures. (a-1) – (d-1) Corresponding line-profiles of the corresponding samples; Figure S8: Surface morphology evolution of AuPd HyCoS NPs with 5 nm Au coating. (a) – (d) AFM top-views of AuPd HyCoS NPs. (a-1) – (d-1) Corresponding zoom-in AFM top-views. (a-2) – (d-2) Cross-sectional line-profiles on the primary NPs. (b-3) – (d-3) Cross-sectional line-profiles on background Au NPs. (e) Summary plot of surface area ratio (SAR) and RMS roughness (Rq) of the hybrid NPs; Figure S9: (a) – (d) EDS spectra of AuPd HyCoS NPs with 5 nm Au coating at different temperatures. (a-1) – (d-1) Zoom-in spectra of Au and Pd. (a-2) – (d-2) EDS elemental composition table for Au and Pd NPs with varying annealing temperature; Figure S10: (a) – (c) Extinction, reflectance, and transmittance spectra of AuPd HyCoS NPs with 5 nm Au coating at different temperatures. (a-1) – (c-2) Normalized and magnified extinction spectra showing peaks and dips along with the different annealing temperature; Figure S11: (a) – (d) AFM side-view (3 × 3 µm2) of AuPd alloyed NPs fabricated with10 nm Au deposited on the 10 nm Pd NP template with different annealing temperature. (a-1) - (d-1) Corresponding cross-sectional line-profile; Figure S12: Surface morphology of AuPd alloyed NPs fabricated with 10 nm Au coating on the 10 nm Pd template at different annealing temperatures. (a) – (d) AFM (3 × 3 mm2) top-views. (a-1) – (d-1) AFM side-views (700 × 700 nm2). (a-2) – (d-2) Cross-sectional line-profiles for corresponding samples. (e) Schematic representation of fabrication of AuPd alloyed NPs. (f) Rq and SAR plots for the corresponding samples. (g) Atomic percentage of Pd and Au for annealed samples; Figure S13: (a) – (a-4) and (c) – (c-4) SEM images and EDS elemental maps for Pd, Au, Al, and O for the AuPd alloy NPs fabricated at 600 and 800 °C respectively. (b) and (d) EDS line-profiles for Au and Pd; Figure S14: (a) – (d) EDS spectra of AuPd alloy NPs with 10 nm Au coating at different temperatures. (a-1) – (d-1) Zoom-in spectra of Au and Pd. (a-2) – (d-2) EDS elemental composition table for Au and Pd NPs with varying annealing temperature; Figure S15: (a) – (c) Extinction (E), reflectance (R), and transmittance (T) spectra of the AuPd alloy NPs. (a-1) Normalized E spectrum. (a-2) Contour plot of extinction peaks. (d) E-field profile side-view of alloyed AuPd NPs; Figure S16: (a) – (e) FDTD schematic for pure Pd, Au, background (BG) Au, alloyed AuPd and HyCoS AuPd NPs. (a-1) – (e-1) Corresponding top-view of e-field profile. (a-2) – (e-2) Corresponding side-view of e-field profile; Figure S17: (a) AFM image of MoS2 nanoplates deposition on bare sapphire. (a-1) Corresponding line-profile. (b) EDS spectrum of MoS2 nanoplates on sapphire. (c) – (c-1) Raman spectra of MoS2, where one drop corresponds to 20 μl of 0.25 mg/ml MoS2 nanoplate solution; Figure S18: (a) – (b) SERS spectra of 2:1 mixture of MoS2 nanoplatelets and R6G at 10-7 M and 10-8 M on HyCoS AuPd NPs. (a-1) – (b-1) Intensity counts of characteristic peaks; Figure S19: (a) Raman spectrum of R6G on bare sapphire. (a-1) Corresponding intensity plots; Figure S20: (a) Liner trend of the SERS signal at 1360 cm-1 at concentration ranging from 10-8 to 10-6 M with the 2:1 mixture MoS2 and 10-6 M R6G on AuPd HyCoS NPs; Table S1: Summary table of SERS peak counts for 10-6 M R6G on various substrates. Counts are in thousand, i.e., × 103. Related to Figure 4(b); Table S2: Enhancement factor (EF) of 10-6 M R6G on MoS2, HyCoS AuPd and mixture solutions (MoS2 : R6G) on AuPd HyCoS NPs at different peak positions; Table S3: Summary table of SERS peak counts of R6G molarity variation on HyCoS AuPd NPs at different peak positions. Counts are in thousand, i.e., × 103. Related to Figure 4(d); Table S4: Summary table of SERS peak counts of various mixture solutions (MoS2 : 10-6 M R6G) on HyCoS AuPdNP substrate at different peak positions. Volume ratios for a total of 20 µl with 0.25 mg/ml MoS2 nanoplatelet concentration. Counts are in thousand, i.e., × 103. Related to Figure 4(f); Table S5: Summary table of SERS peak counts of 10-7 and 10-8¬ M R6G with and without MoS2 on HyCoS AuPd NPs. 2:1 (MoS2 : R6G). Counts are in thousand, i.e., × 103. Related to Figure S17. References [12,27,28,29,33,48,55,56,57,58,59,60] are cited in the supplementary materials.
