# Supplementary material for: MoS2 Nanoplatelets on Hybrid Core-Shell (HyCoS) AuPd NPs for Hybrid SERS Platform for Detection of R6G
Source: Nanomaterials (Basel). 2023 Feb 18;13(4):769. doi: 10.3390/nano13040769 (PMC9963033; doi:10.3390/nano13040769)
Supplement: Supplementary file 1 [file nanomaterials-13-00769-s001.zip › nanomaterials-2207047-supplementary.pdf]

# Supplementary

## $MoS_2$ nanoplatelets on hybrid core-shell (HyCoS) AuPd NPs for hybrid SERS platform for detection of R6G

Shusen Lin[1], Rutuja Mandavkar[1], Shalmali Burse[1],Md Ahasan Habib[1], Tasmia Khalid[1], Mehedi Hasan Joni[1], Sundar Kunwar[1,2*] and Jihoon Lee[1]**

[1] Department of Electronic Engineering, College of Electronics and Information, Kwangwoon University, Nowon-gu, Seoul, 01897, South Korea.

[2] Center for Integrated Nanotechnologies (CINT), Los Alamos National Laboratory, Los Alamos, New Mexico 87545, USA

Correspondence e-mail: sundar@lanl.gov (S. Kunwar)*, jihoonlee@kw.ac.kr (J. Lee)**

## S.1 Detailed experimental section:

### S.1.1. Preparation of substrate for hybrid core shell (HyCoS) AuPd NP fabrication

A double-side polished c-plane sapphire (0001) wafers with a thickness of 430 µm and ± 0.1° off-axis (INexus Inc., South Korea) were used for the fabrication of AuPd hybrid core-shell (HyCoS) NPs. Sapphire substrates were adapted due to its superior physical and chemical stability and low surface energy [12,33]. To begin with, the sapphire wafers were degassed in the pulsed laser deposition (PLD) vacuum chamber below $1.0 \times 10^{-4}$ Torr at 600 °C for 30 min to induce the removal of trapped particulates, oxides, and water vapors [12,33]. The surface morphology after the vacuum-thermal-degassing is provided in Fig. S1, exhibiting the surface roughness less than 1 nm. The average transmittance and reflectance of degassed sapphires were ~15 % and 84 %.

### S.1.2. Fabrication of Pd NP template and various plasmonic AuPd NPs by droplet epitaxy

*Pd NPs template:* 10 nm Pd film was deposited on the degassed sapphire by ion-coater under vacuum condition from the Pd target of 99.999 % purity. The ionization current and base pressure were 3 mA and $1 \times 10^{-1}$ Torr respectively, demonstrating the deposition rate of 0.05 nm $s^{-1}$. Subsequently, the droplet epitaxy through solid-state dewetting (SSD) process was conducted in PLD chamber at an annealing temperature of 800 °C for 450 s. During the droplet epitaxy process, Pt droplets were formed by the diffusion of Pd adatoms assisted by thermal energy. The well-developed dome-shaped Pd NPs were formed on sapphire and the corresponding morphological and optical characterizations are provided in Fig. S2.

*Plasmonic AuPd NPs:* Various thicknesses of Au films such as 3, 5 and 10 nm were sputtered on the as-prepared Pd NP template by using Au metal targets with a purity of 99.999 %. Then, the 2$^{nd}$ step droplet epitaxy was carried out in the PLD chamber at various annealing temperatures such as 400, 600 and 800 °C. The target temperature was attained at a 4 °C/s ramping rate, which was controlled by

a computer recipe program. Various AuPd NP substrates were taken out after terminating the droplet epitaxy and cooling down to room temperature.

### S.1.3. Preparation of R6G, $MoS_2$ nanoplatelets and mixture solutions

Rhodamine 6G (R6G, Sigma Aldrich) was dissolved in ethanol (Sigma Aldrich) to make $10^{-3}$ M stock solution and then the prepared solution was further diluted to prepare various molarity of R6G from $10^{-4}$ to $10^{-8}$ M concentrations. Molybdenum disulfide ($MoS_2$, Sigma Aldrich) nanoplatelet powder was dissolved in ethanol to make 0.25 mg $ml^{-1}$. The mixture solutions were prepared by mixing the R6G and $MoS_2$ at various ratios of 1:1, 2:1 and 10:1. A total of 20 μl of various solutions such as R6G and mixture solutions were drop-casted on the AuPd substrate for SERS detection.

Here, the $MoS_2$ nanoplatelets were 50 ~ 1000 nm in diameter with thickness < 3 layers. $MoS_2$ nanoplatelet is a two-dimensional (2-D) layered transition metal dichalcogenide (TMD) material.[27,29] Due to the unique thickness-dependent physical and chemical properties, the $MoS_2$ has drawn significant attention in many fields such as chemical catalysts, biochemical sensing, optical sensors and SERS [27,28]. Owing to the existence of covalent Mo and S bonds with numerous absorption edges, superior molecular interactions and charge transfer has been demonstrated, which has a great impact on the Raman signal amplification.[27,29] The characterization of $MoS_2$ nanoplatelets on bare sapphire can be found in Fig. S17. Fig. S17(a) shows the top-view of AFM image and the average height of $MoS_2$ nanoplatelet deposition was found to be ~ 10 nm. In Fig. S17(b), Mo Lα1 and S Kα peaks are clearly pronounced in the EDS spectrum. Fig. S17(c) shows the Raman spectrum of $MoS_2$ nanoplatelets. The typical $E_{2g}^1$ and $A_{1g}$ vibration modes of $MoS_2$ were clearly seen at 380 and 405 $cm^{-1}$ in Fig. S17(c-1) [55]. The gradually increased $MoS_2$ peak intensity with the additional depositions due to the increased scattering is clearly seen in Fig. S17(c-1) [7].

### S.1.4. Morphological and optical characterizations

Atomic force microscope (AFM) (XE-70, Park Systems Corp., South Korea) was used for the surface morphological analysis of the fabricated HyCoS NPs. Energy-dispersive x-ray spectroscope (EDS) systems (Noran System 7, Thermo Fisher, United States) (Ultimax, Oxford Instruments, United Kingdom) were utilized for the elemental analyses. The optical characteristics of fabricated HyCoS NPs were obtained by a NOST system (Nostoptiks, South Korea), equipped with an ANDOR sr-500i spectrograph, CCD detector, and combined halogen-deuterium (200 ~ 1100 nm) light sources. The optical spectra, namely reflectance and transmittance, were experimentally obtained whereas the extinction spectra were derived by using the relation: extinction (%) + reflectance (%) + transmittance (%) = 100%. The Raman measurements were performed with the Raman spectrometer UNIRAM II (UniNanoTech, South Korea) with an excitation laser of 532 nm in backscattering geometry. The spot size of laser was less than 1 μm with 100× objective lens and the power at the sample surface was 9 mW. The grating of 1200 gr./mm was used to record the spectra in the wavenumber region between 500 and 1700 $cm^{-1}$ using a CCD detector. The slit width was set at 200 nm and the samples were measured with the expose time of 0.8 sec with 10 accumulations.

**S.1.5. Finite difference time domain (FDTD) simulation**

To probe the LSPR properties and the distribution of the local e-field of various metallic NPs, finite difference time domain (FDTD) simulation (Lumerical Solutions, Canada) was adapted. The total-field scattered-field (TFSF) source was employed to analyze the scattering of nanoparticles (NPs). The TFSF source separates the computation region into the total field and scattered field regions. The scattering and absorption monitors were used to determine the excitation power, which is based on excitation = absorption + scattering. The DFT monitors were placed in the same dimension to study the near e-filed profile and to avoid the difference in the spectrum. The perfectly matched layer (PML) boundary and the nanostructure were designed to allow radiation propagation out of the computational regions without disturbing the fields inside. The PML boundary absorbs the electromagnetic wave and

demonstrated more effectiveness for radiation absorption. The mesh size with 0.5 nm in X, Y and Z was used to improve the accuracy. The refractive indicies of sapphire and Au were fitted by the Palik model and the refractive index for Pd was fitted by the Palm model [56,57]. The refractive index of $MoS_2$ is fitted by Beal and Hughes Model [58]. The top- and side-views of e-field distribution were summarized in Fig. S16, where the calculated results showed a good agreement with the experimental data.

**S.1.6. SERS enhancement factor (EF) calculation**

The SERS enhancement factor (EF) was calculated using the following equation [7,59];

$$EF = \frac{I_{SERS} N_{Raman}}{I_{Raman}\, N_{SERS}} = \frac{I_{SERS}}{I_{Raman}} \times \frac{\rho \times h \times S_{laser} \times \left(\frac{Na}{\mathrm{M}}\right)}{S_{laser} \,/\, S_{probe\ molecule}} \quad (1)$$

$I_{SERS}/I_{Raman}$ is the intensity ratio of SERS and Raman, $\rho$ is the density of probe molecules, $h$ is the effective layer depth and M is the molar mass of probe molecules. Na is the Avogadro constant with the value of $6.022 \times 10^{23}$. $S_{laser}$ is the laser spot size and $S_{probe\ molecule}$ is the surface area of probe molecules. Here, the $S_{laser}$ is the spot size of laser, i.e., $1 \times 10^{-4}$ $cm^2$ in this work. The effective layer depth ($h$) of drop-casted R6G solution was considered to be $3.8 \times 10^{-3}$ cm.[7] The density ($\rho$) of R6G is 1.26 $g/cm^3$ and the molar mass is 479 g/mol. $S_{R6G}$ is 61.5 $Å^2$. [https://pubchem.ncbi.nlm.nih.gov/compound/Rhodamine-6G] The Raman signal of $10^{-3}$ M R6G on bare sapphire is shown in Fig. S18. To obtain Raman signal of $10^{-3}$ M R6G, 20 µl $10^{-3}$ M R6G was applied on bare sapphire and the Raman signal was measured under the same measurement condition, exhibiting weak signals of characteristic peaks of R6G. The measurement parameters are same to those used in SERS with 1 µm spot size, 0.8 s exposure time, 10 times accumulation and 200 µm slit width. The normal Raman intensity ($I_{Raman}$) at $10^{-6}$ M was obtained by dividing by $10^3$. The calculated characteristic peak-counts ($I_{Raman}$) of $10^{-6}$ M R6G on bare sapphire are $2.26 \times 10^{-4}$, $2.30 \times 10^{-4}$, $3.35 \times 10^{-4}$, $2.75 \times 10^{-4}$ and $3.42 \times 10^{-4}$ at 612, 770, 1360, 1506, 1648 $cm^{-1}$.

### S.1.7. Limit of detection (LOD) calculation

The LOD indicates the lowest concentration at which the SERS signals can be distinguish from the background noise [48,60]. The LOD is determined by the following equation:[60]

$$LOD = \frac{3\sigma}{b} \quad (2)$$

where σ is the residual standard deviation and b is the slope of regression line. To determine the limit of detection (LOD) of hybrid platform, the liner relationship in the range between $10^{-8}$ and $10^{-6}$ M was utilized as presented in Fig. S20. The peak at 1360 $cm^{-1}$ with the highest EF was used. σ is determined by the following equation:[48]

$$\sigma = \sqrt{\frac{\sum_{i=1}^{n}(x_i - \bar{x})^2}{(n-1)}} \quad (3)$$

Where the $x_i$ is the measurement value of background (blank) response and $\bar{x}$ is the mean value of the background response on bare sapphire, which can be calculated as

$$\bar{x} = \frac{\sum_{i=1}^{n} x_i}{n} \quad (4)$$

The $\sigma$ was obtained to be 15.90 with the 10-time blank readings. In this case, the LOD of hybrid platform was calculated to be $6.68 \times 10^{-10}$ M.

## Bare sapphire

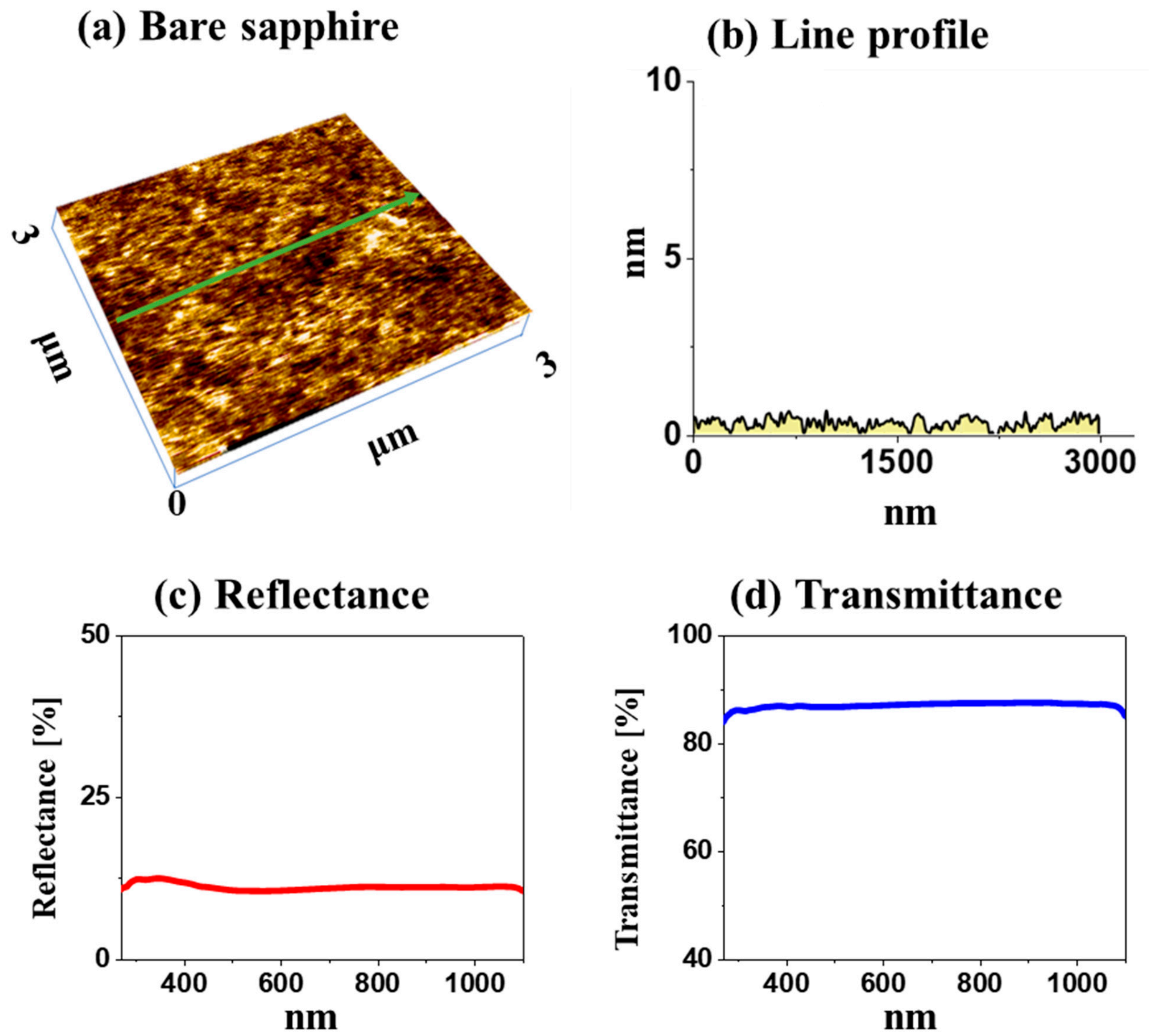


**Figure S1:** (a) Atomic Force Microscopy (AFM) side-view of bare sapphire (0001) degassed at 600 °C for 30 min. (b) Corresponding surface line-profile. (c) Transmittance spectra with ~ 84 % average transmittance. (d) Reflectance spectra with 13 % average reflectance.

## Pd NPs on sapphire

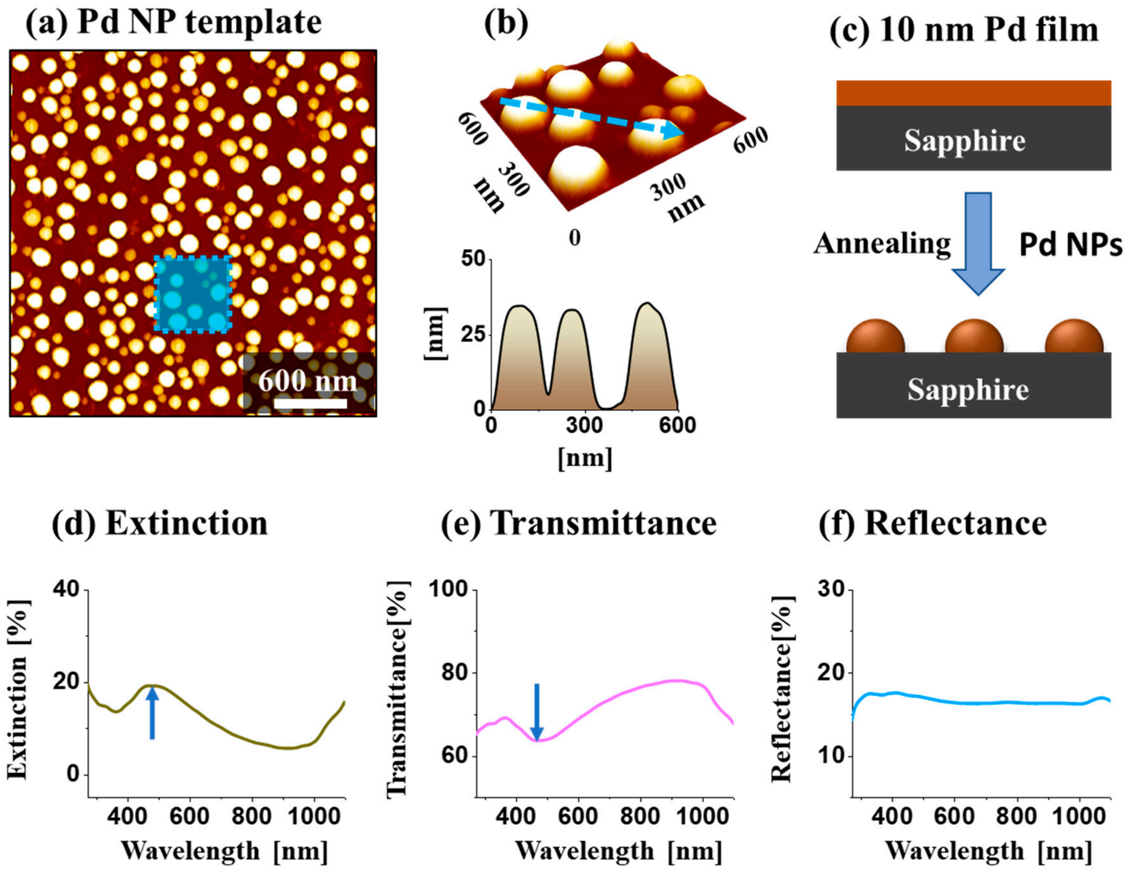


**Figure S2:** Fabrication of palladium (Pd) nanoparticles (NPs) by droplet epitaxy of 10 nm Pt film on sapphire (0001) at 800 °C for 450 s. (a) Large scale AFM top-view (3 × 3 μm$^2$). (b) AFM side-view with corresponding line-profile. (c) Schematic representation of Pd NP formation by droplet epitaxy. (d) – (f) Extinction, transmittance and reflectance spectra of Pd template.

**- Evolution of various AuPd NPs and their characteristics**

a. **HyCoS AuPd NPs with 3 nm Au coating**

b. **HyCoS AuPd NPs with 5 nm Au coating**

c. **Alloy AuPd NPs with 10 nm Au coating**

# HyCoS AuPd NPs coated with 3 nm Au

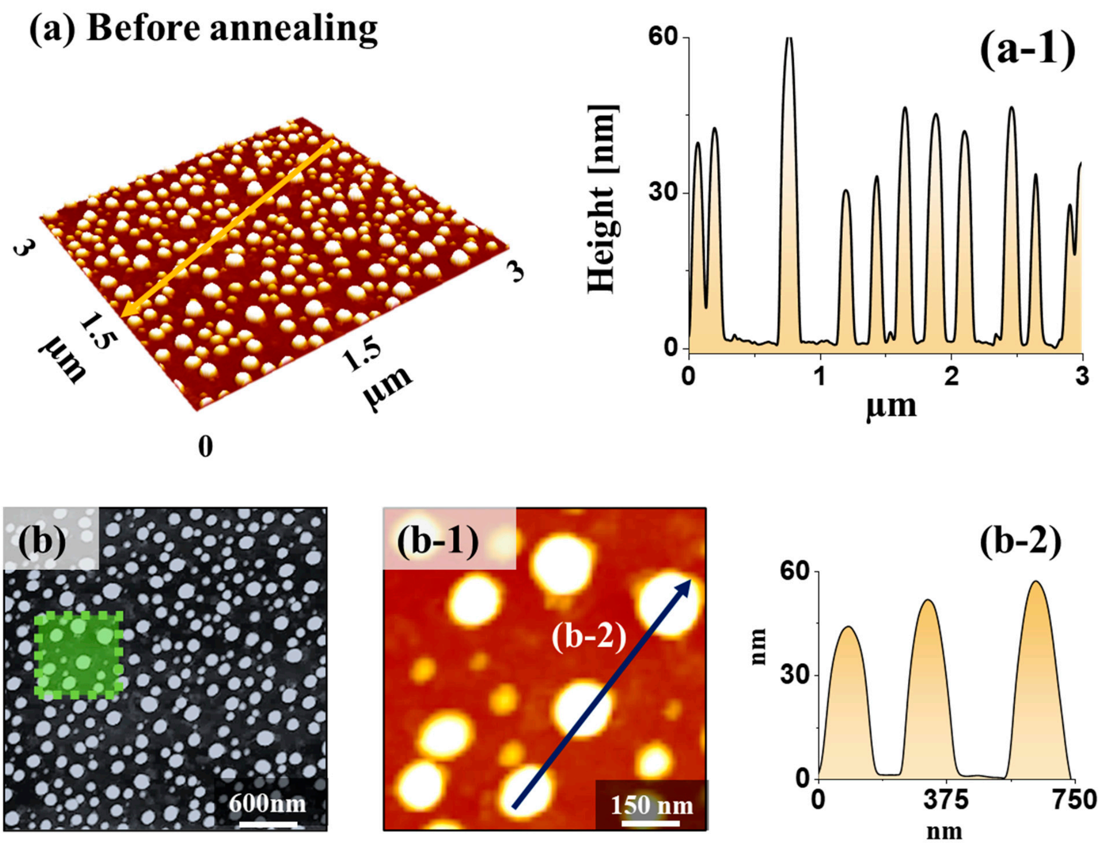


**Figure S3:** Surface morphological characterizations of AuPd NPs with 3 nm Au coating before annealing. (a) – (a-1) AFM side-views (3 × 3 $\mu m^2$) and corresponding line-profile. (b-1) – (b-2) AFM top-view, enlarged top-view and corresponding line-profile.

# HyCoS AuPd NPs coated with 3 nm Au

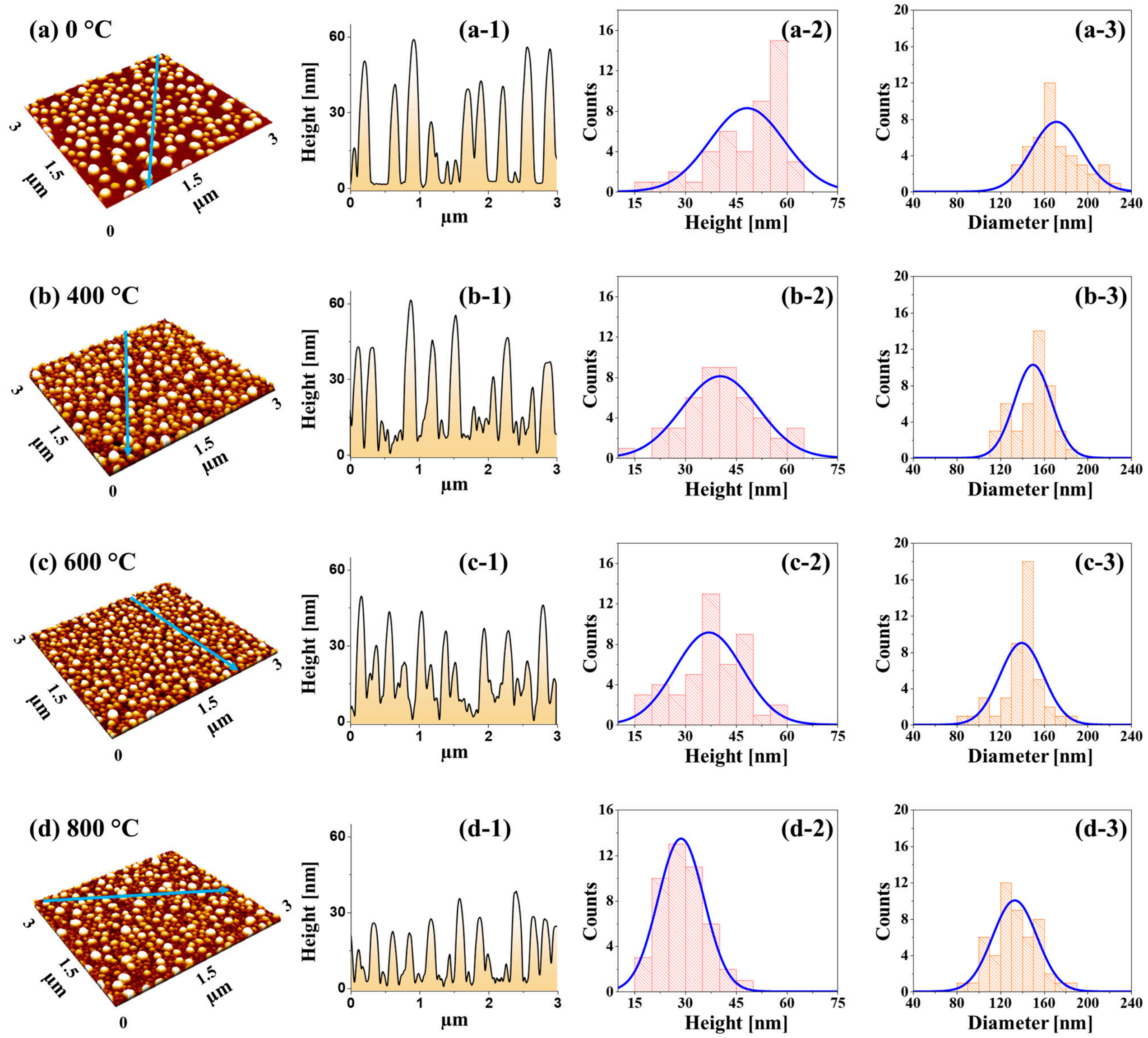


**Figure S4:** (a) – (c) AFM side-views (3 × 3 μm$^2$) of AuPd hybrid core-shell (HyCoS) NPs with 3 nm Au coating annealed at various temperatures. (a-1) – (c-1) Corresponding cross-sectional line-profiles. (a-2) – (d-2) Height distribution histograms of core-shell AuPd NPs. (a-3) – (d-3) Diameter distribution histograms of core-shell AuPd NPs.

# HyCoS AuPd NPs coated with 3 nm Au

(a) 0 °C

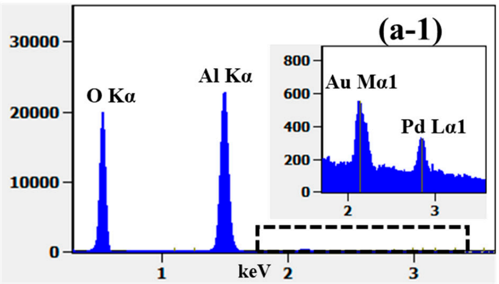


(a-2)

| ElementZ | Wt % | At % |
|---|---|---|
| Au M | 67.19 | 52.22 |
| Pd L | 32.81 | 47.48 |

(b) 400 °C

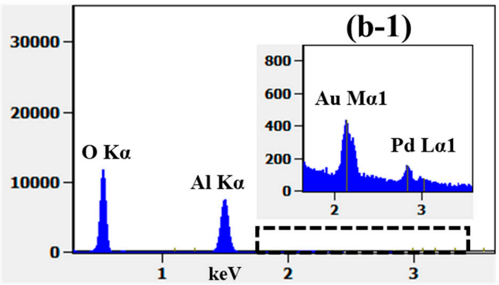


(b-2)

| Element | Wt % | At % |
|---|---|---|
| Au M | 60.71 | 45.50 |
| Pd L | 39.29 | 54.50 |

(c) 600°C

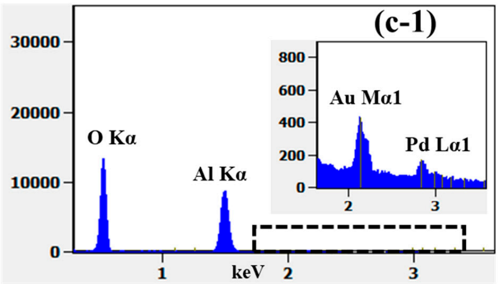


(c-2)

| Element | Wt % | At % |
|---|---|---|
| Au M | 47.46 | 32.79 |
| Pd L | 52.54 | 67.21 |

(d) 800°C

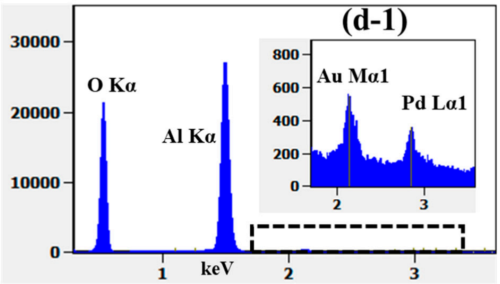


(d-2)

| Element | Wt % | At % |
|---|---|---|
| Au M | 42.17 | 28.26 |
| Pd L | 57.83 | 71.74 |

**Figure S5:** (a) – (d) EDS spectra of AuPd HyCoS NPs with 3 nm Au coating at different temperatures. (a-1) – (d-1) Zoom-in spectra of Au and Pd. (a-2) – (d-2) EDS elemental composition table for Au and Pd NPs with varying annealing temperature.

# HyCoS AuPd NPs coated with 3 nm Au

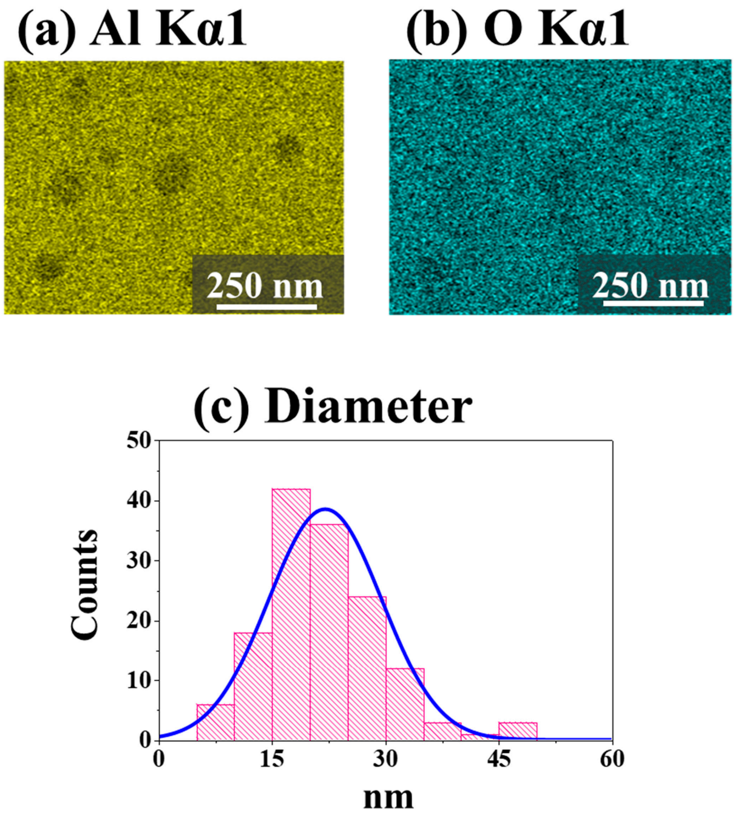


**Figure S6:** (a) – (b) EDS phase maps of Al and O. Related to Fig. 2(g). (c) Diameter distribution histogram of background Au NPs on bare sapphire.

# HyCoS AuPd NPs coated with 5 nm Au

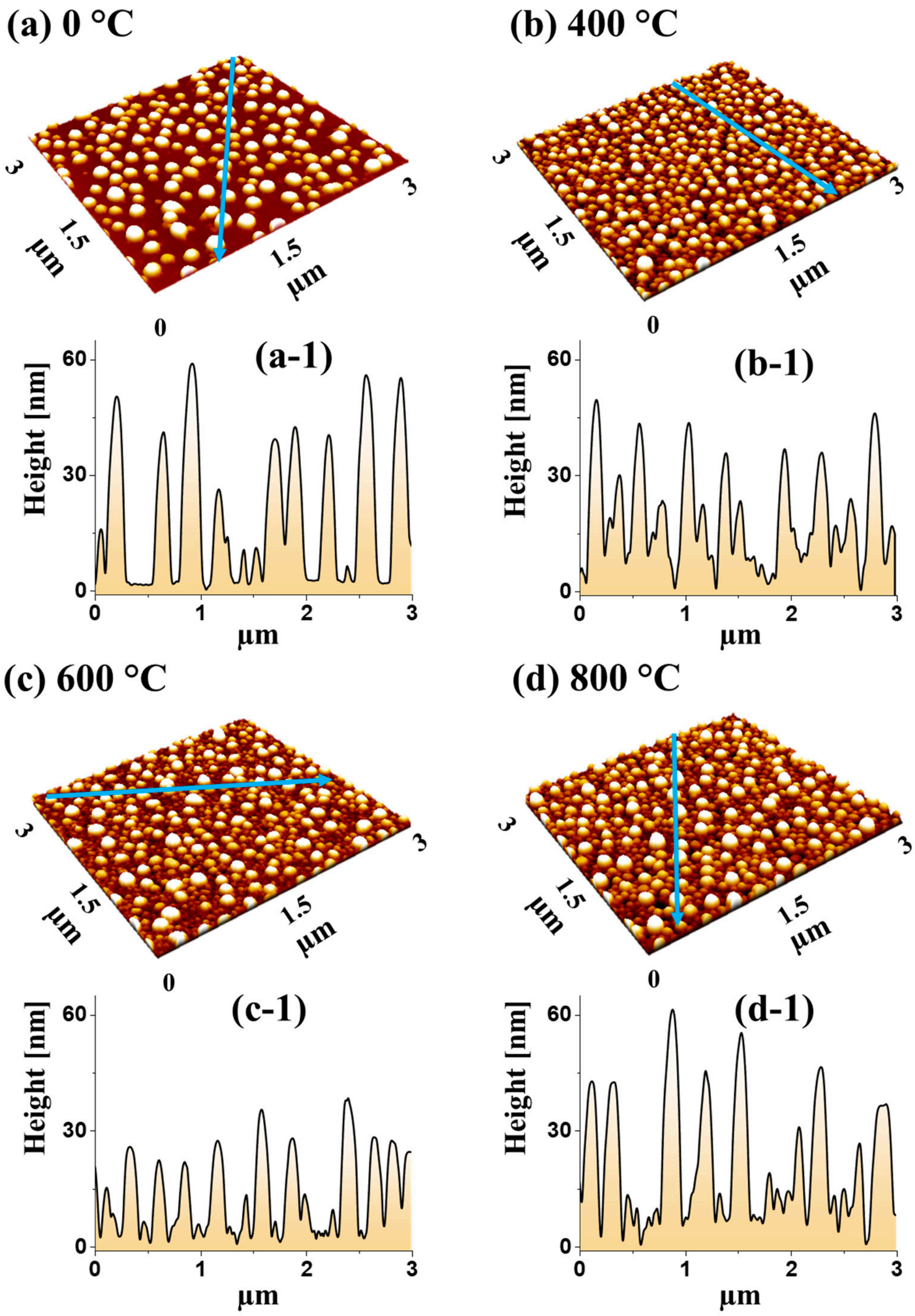


**Figure S7:** (a) – (d) AFM side-views (3 × 3 $\mu m^2$) of AuPd HyCoS NPs with 5 nm Au coating annealed at various temperatures. (a-1) – (d-1) Corresponding line-profiles of the corresponding samples.

# HyCoS AuPd NPs coated with 5 nm Au

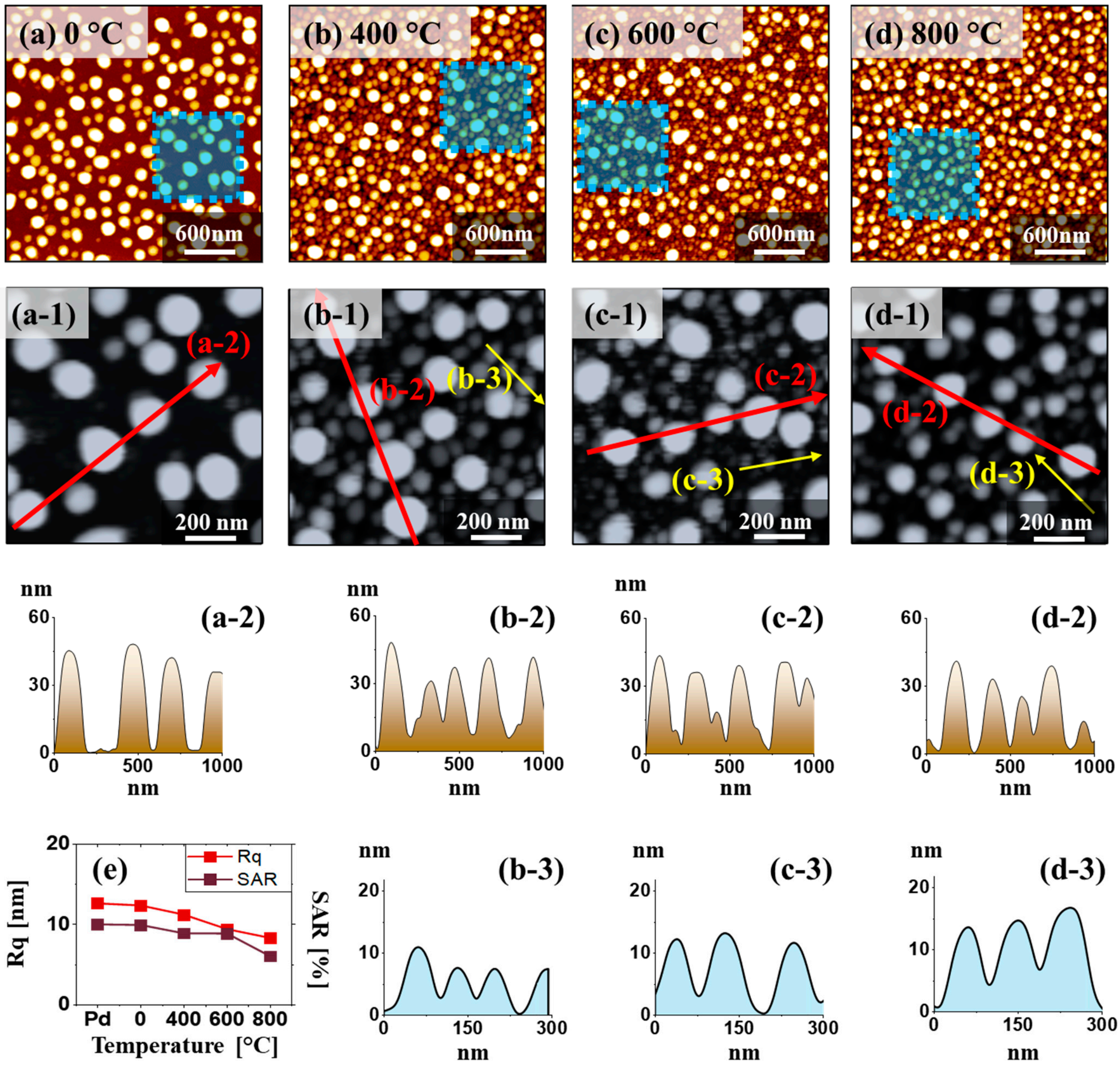


**Figure S8:** Surface morphology evolution of AuPd HyCoS NPs with 5 nm Au coating. (a) – (d) AFM top-views of AuPd HyCoS NPs. (a-1) – (d-1) Corresponding zoom-in AFM top-views. (a-2) – (d-2) Cross-sectional line-profiles on the primary NPs. (b-3) – (d-3) Cross-sectional line-profiles on background Au NPs. (e) Summary plot of surface area ratio (SAR) and RMS roughness (Rq) of the hybrid NPs.

# HyCoS AuPd NPs coated with 5 nm Au

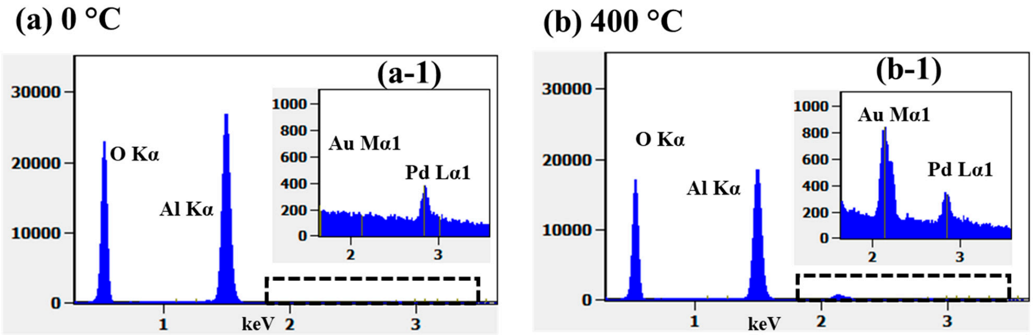


(a-2)

| Element | Wt % | At % |
|---|---|---|
| Au M | 70.08 | 55.86 |
| Pd L | 29.92 | 44.14 |

(b-2)

| Element | Wt % | At % |
|---|---|---|
| Au M | 59.89 | 44.65 |
| Pd L | 40.11 | 55.35 |

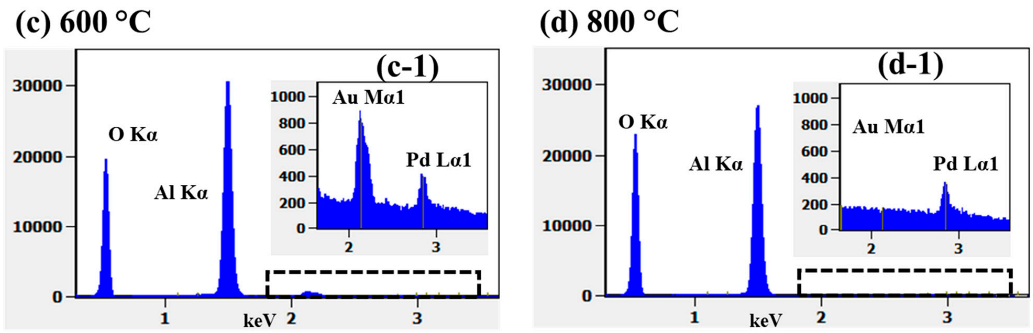


(c-2)

| Element | Wt % | At % |
|---|---|---|
| Au M | 58.46 | 43.19 |
| Pd L | 41.54 | 56.81 |

(d-2)

| Element | Wt % | At % |
|---|---|---|
| Au M | 50.49 | 35.53 |
| Pd L | 49.51 | 64.47 |

**Figure S9:** (a) – (d) EDS spectra of AuPd HyCoS NPs with 5 nm Au coating at different temperatures. (a-1) – (d-1) Zoom-in spectra of Au and Pd. (a-2) – (d-2) EDS elemental composition table for Au and Pd NPs with varying annealing temperature.

## HyCoS AuPd NPs coated with 5 nm Au

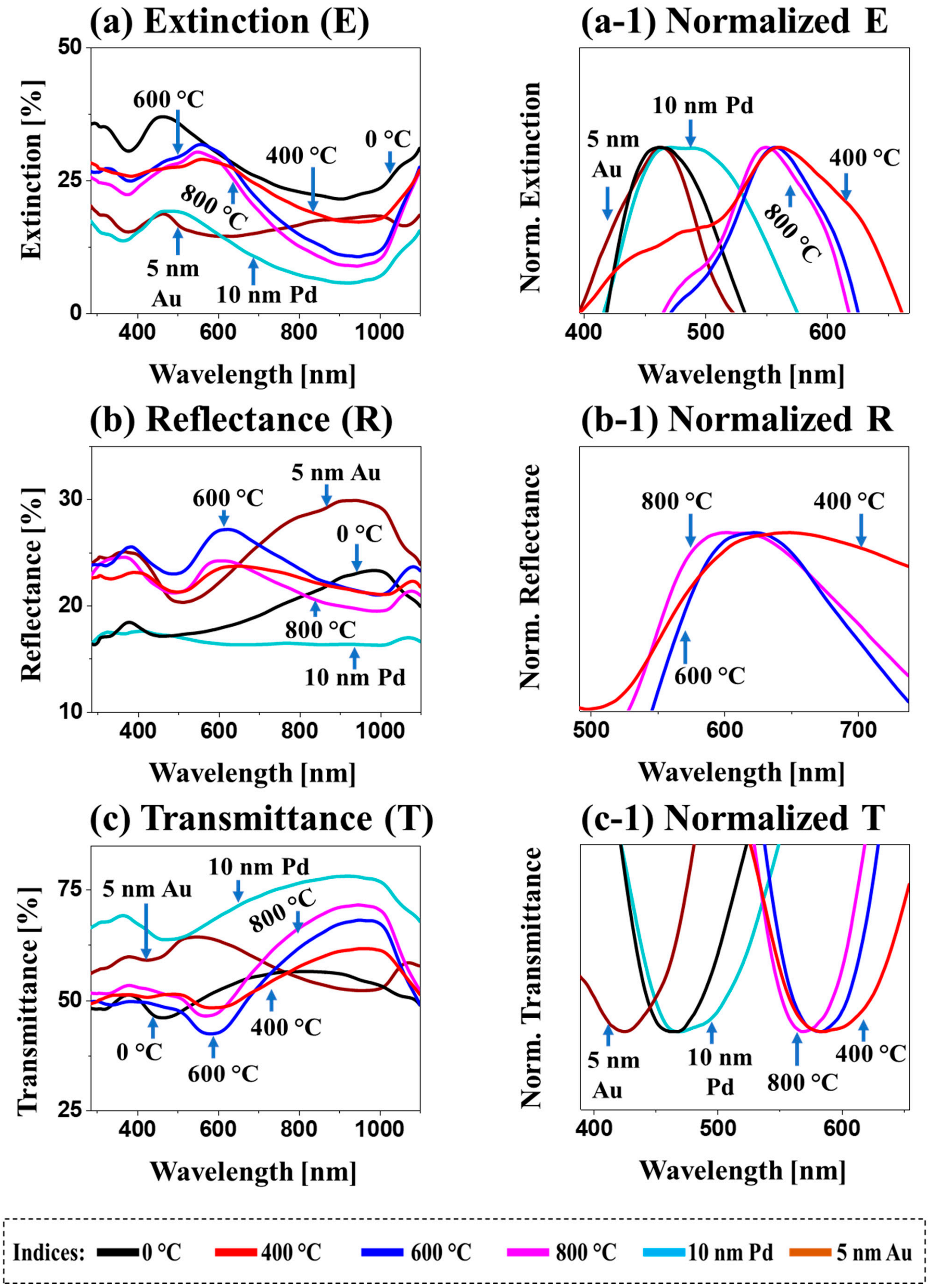


**Figure S10:** (a) – (c) Extinction, reflectance, and transmittance spectra of AuPd HyCoS NPs with 5 nm Au coating at different temperatures. (a-1) – (c-2) Normalized and magnified extinction spectra showing peaks and dips along with the different annealing temperature.

# Alloy AuPd NPs coated with 10 nm Au

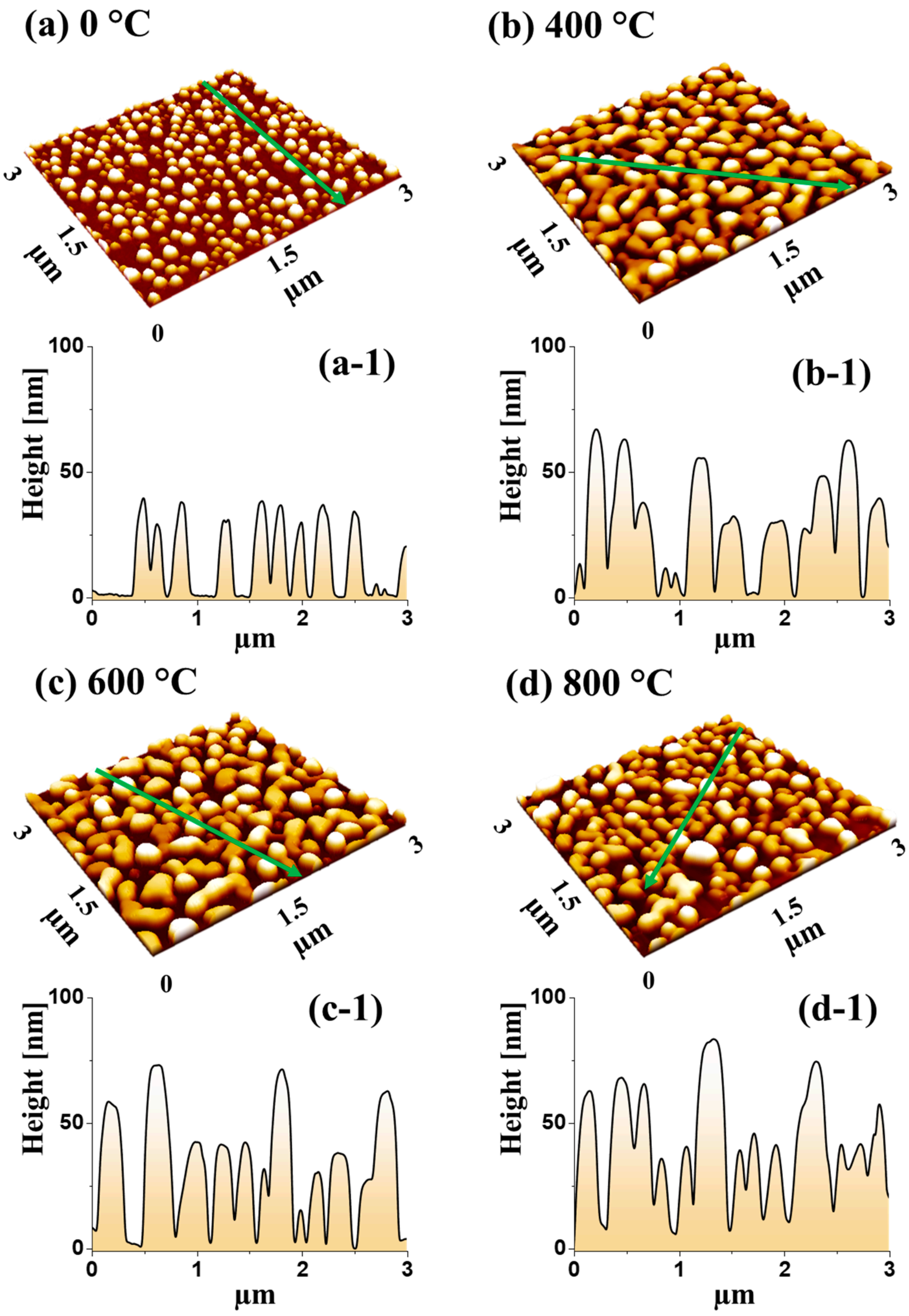


**Figure S11:** (a) – (d) AFM side-view (3 × 3 μm$^2$) of AuPd alloyed NPs fabricated with10 nm Au deposited on the 10 nm Pd NP template with different annealing temperature. (a-1) - (d-1) Corresponding cross-sectional line-profile.

# Alloy AuPd NPs coated with 10 nm Au

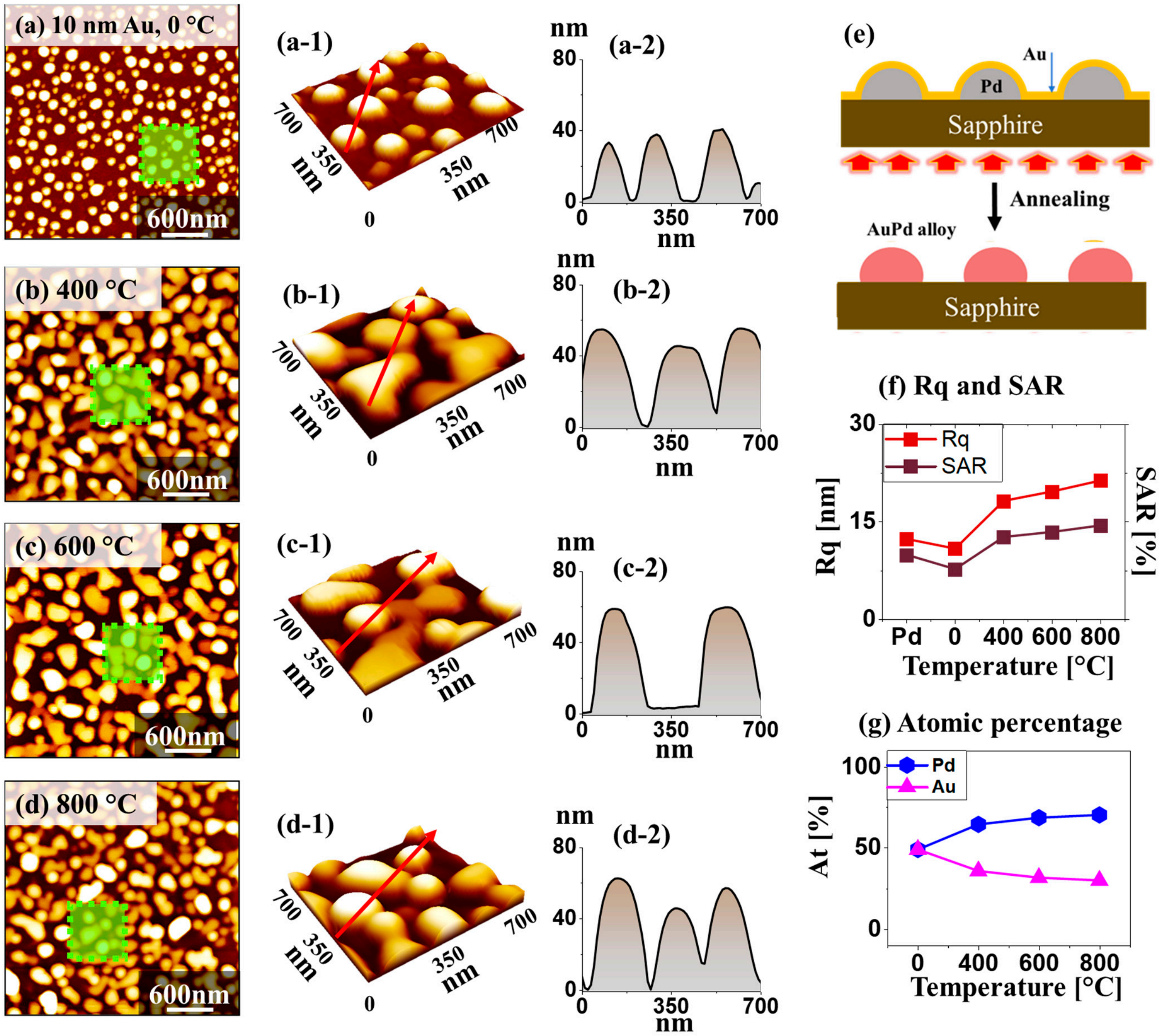


**Figure S12:** Surface morphology of AuPd alloyed NPs fabricated with 10 nm Au coating on the 10 nm Pd template at different annealing temperatures. (a) – (d) AFM (3 × 3 mm$^2$) top-views. (a-1) – (d-1) AFM side-views (700 × 700 nm$^2$). (a-2) – (d-2) Cross-sectional line-profiles for corresponding samples. (e) Schematic representation of fabrication of AuPd alloyed NPs. (f) Rq and SAR plots for the corresponding samples. (g) Atomic percentage of Pd and Au for annealed samples.

## Alloy AuPd NPs coated with 10 nm Au

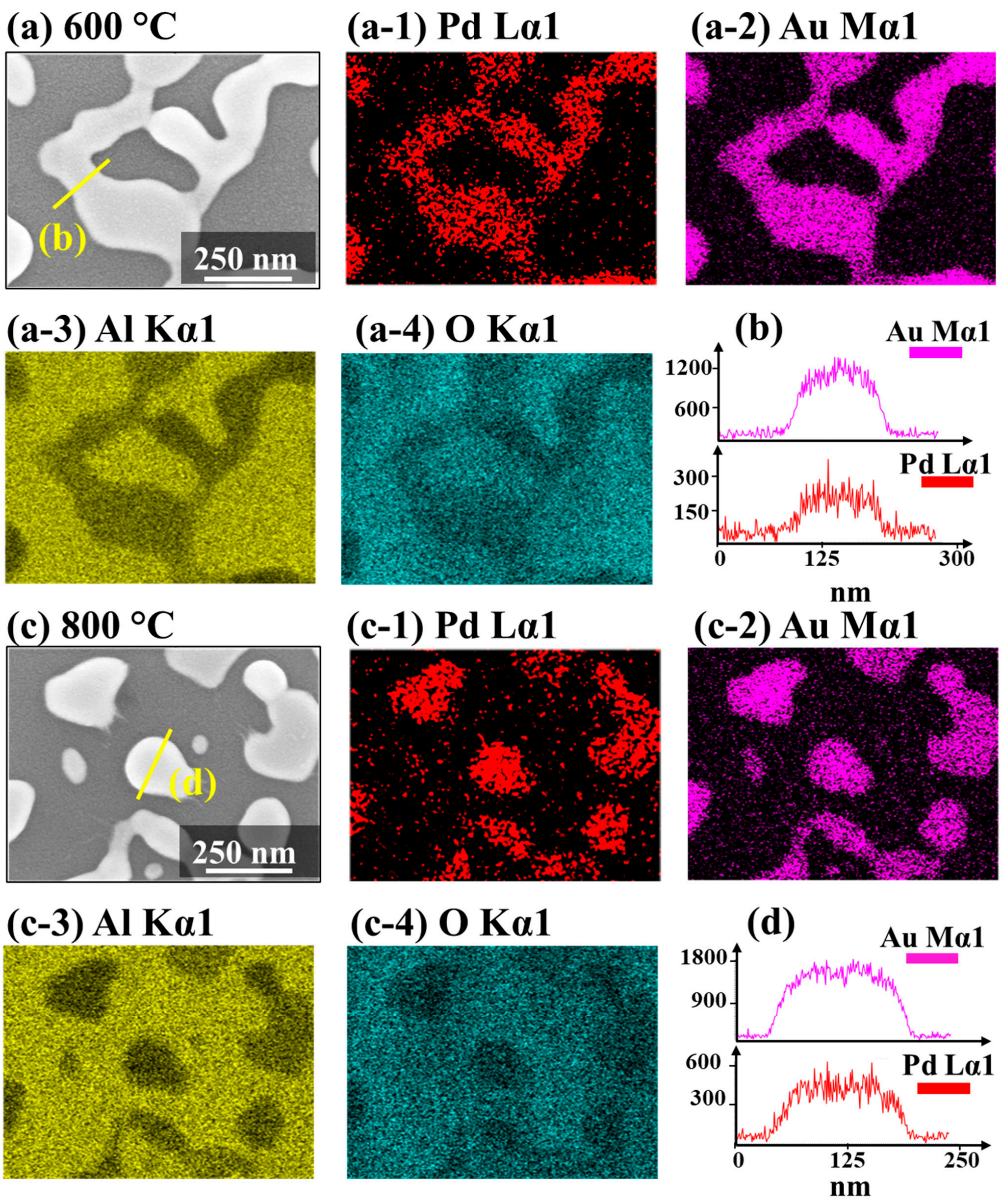


**Figure S13:** (a) – (a-4) and (c) – (c-4) SEM images and EDS elemental maps for Pd, Au, Al, and O for the AuPd alloy NPs fabricated at 600 and 800 °C respectively. (b) and (d) EDS line-profiles for Au and Pd.

# Alloy AuPd NPs coated with 10 nm Au

(a) 0 °C

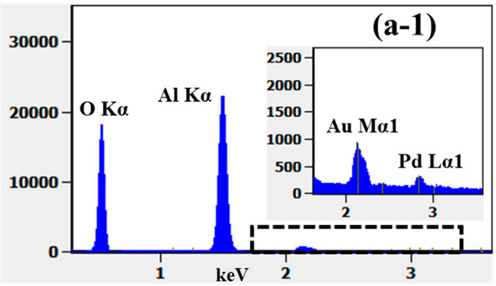


(a-2)

| Element | Wt % | At % |
|---|---|---|
| Au M | 65.93 | 51.11 |
| Pd L | 34.07 | 48.89 |

(b) 400 °C

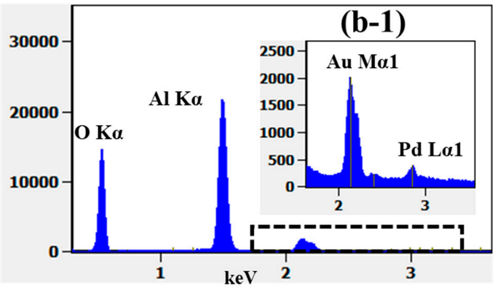


(b-2)

| Element | Wt % | At % |
|---|---|---|
| Au M | 76.94 | 64.32 |
| Pd L | 23.06 | 35.68 |

(c) 600 °C

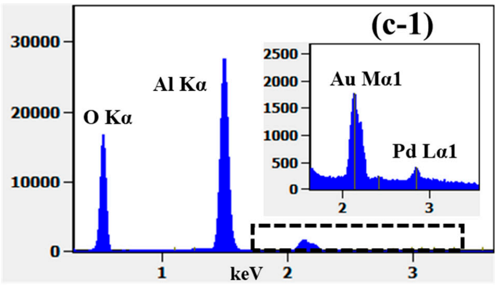


(c-2)

| Element | Wt % | At % |
|---|---|---|
| Au M | 80.06 | 68.44 |
| Pd L | 19.94 | 31.56 |

(d) 800 °C

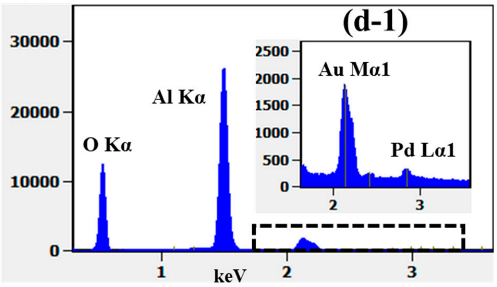


(d-2)

| Element | Wt % | At % |
|---|---|---|
| Au M | 81.30 | 70.14 |
| Pd L | 18.70 | 29.86 |

**Figure S14:** (a) – (d) EDS spectra of AuPd alloy NPs with 10 nm Au coating at different temperatures. (a-1) – (d-1) Zoom-in spectra of Au and Pd. (a-2) – (d-2) EDS elemental composition table for Au and Pd NPs with varying annealing temperature.

# Alloy AuPd NPs coated with 10 nm Au

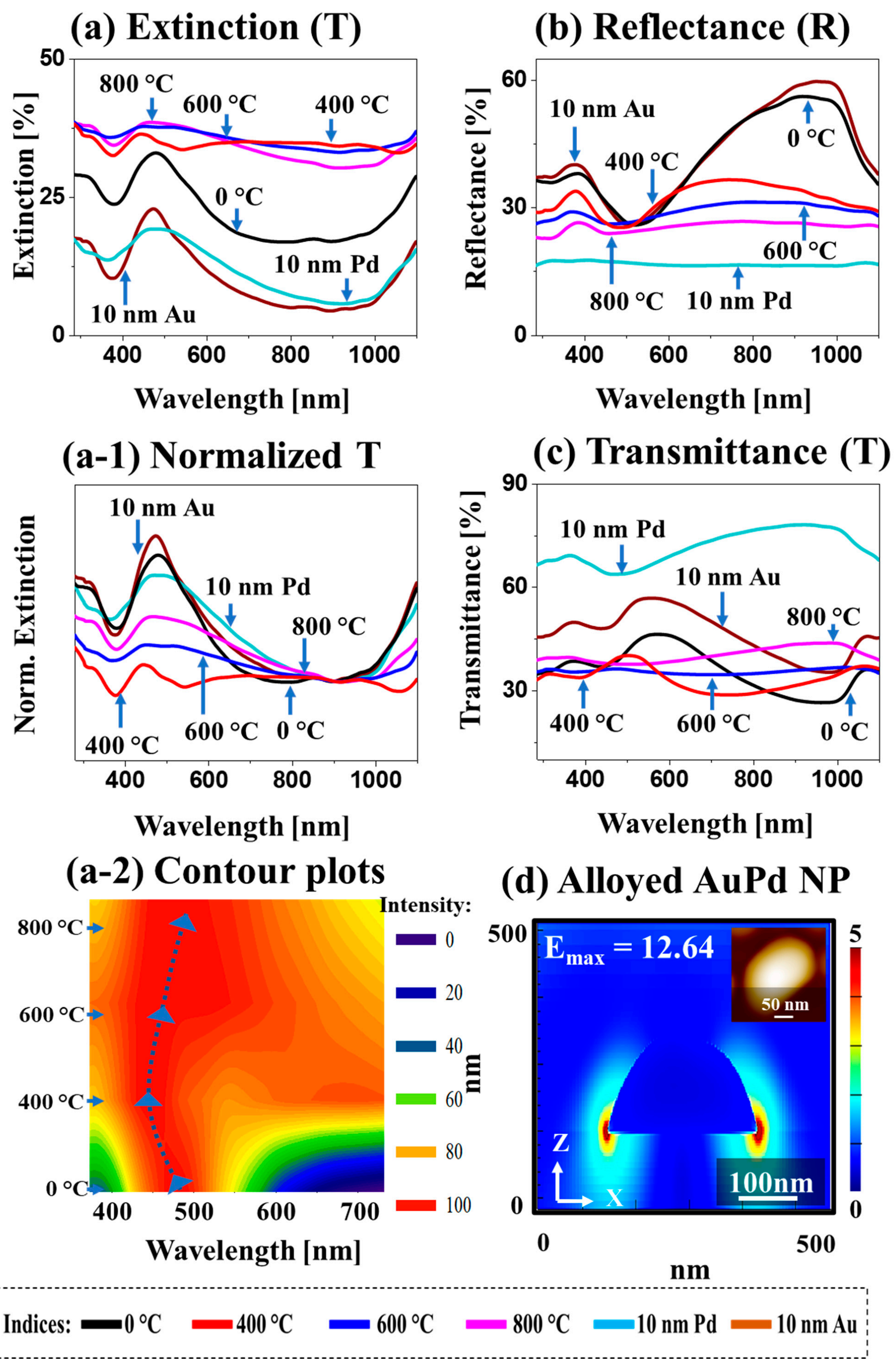


**Figure S15:** (a) – (c) Extinction (E), reflectance (R), and transmittance (T) spectra of the AuPd alloy NPs. (a-1) Normalized E spectrum. (a-2) Contour plot of extinction peaks. (d) E-field profile side-view of alloyed AuPd NPs.

## FDTD simulations

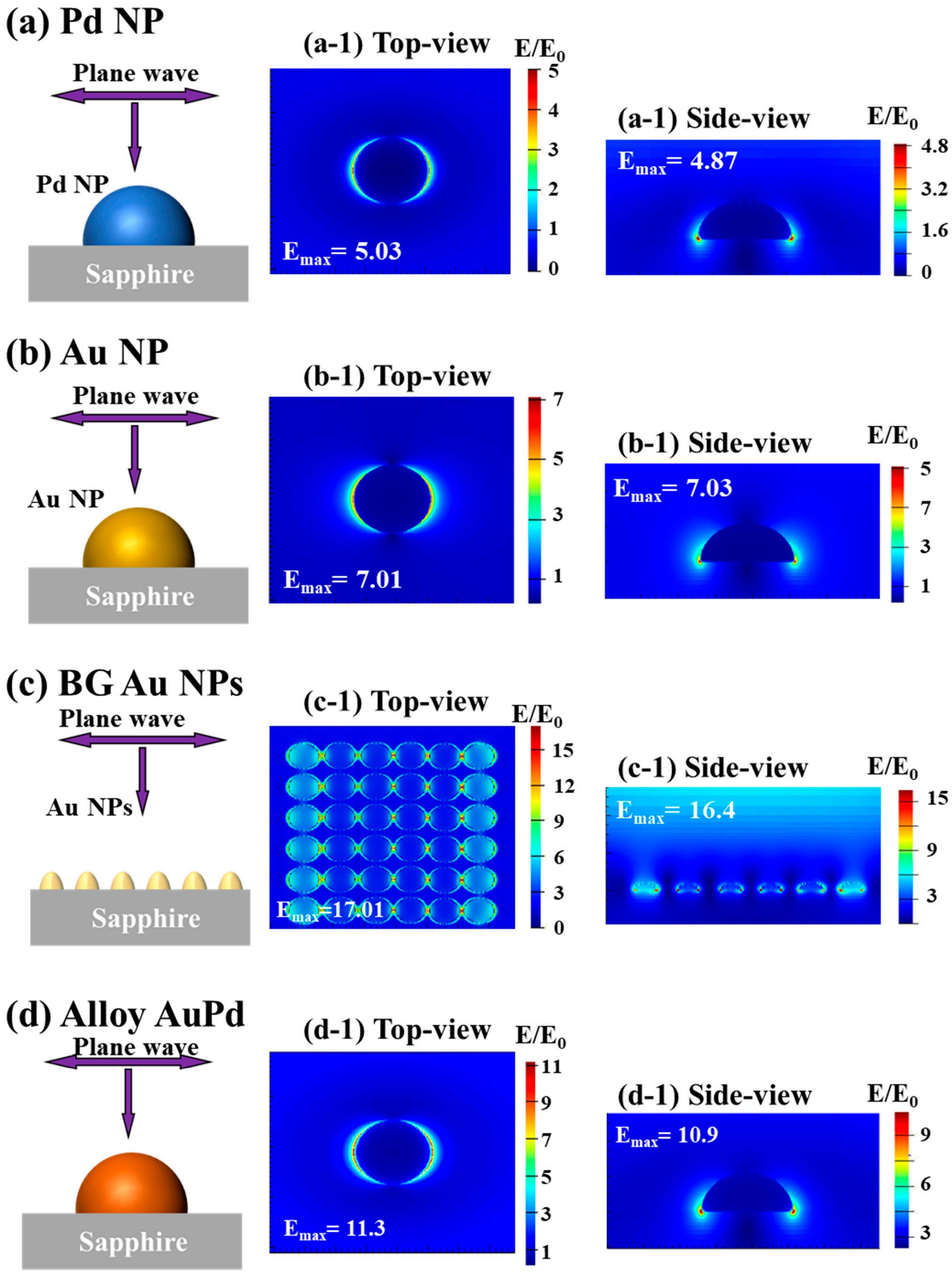

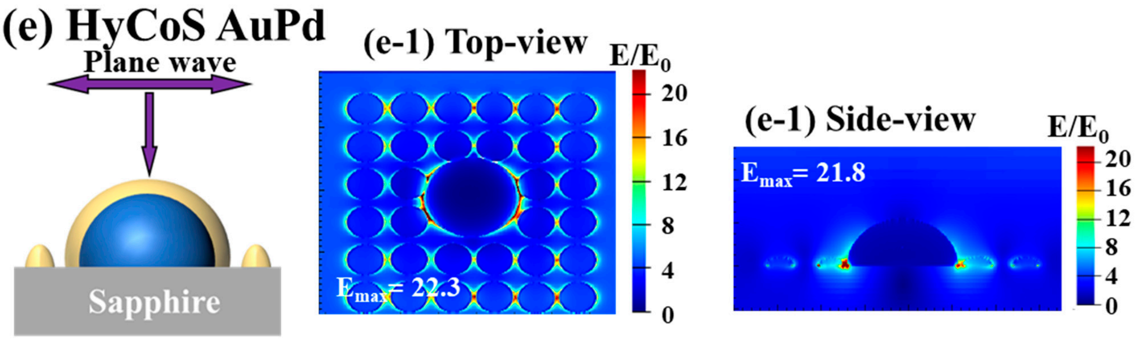


**Figure S16:** (a) – (e) FDTD schematic for pure Pd, Au, background (BG) Au, alloyed AuPd and HyCoS AuPd NPs. (a-1) – (e-1) Corresponding top-view of e-field profile. (a-2) – (e-2) Corresponding side-view of e-field profile.

# Characterization of $MoS_2$ nanoplates on bare sapphire

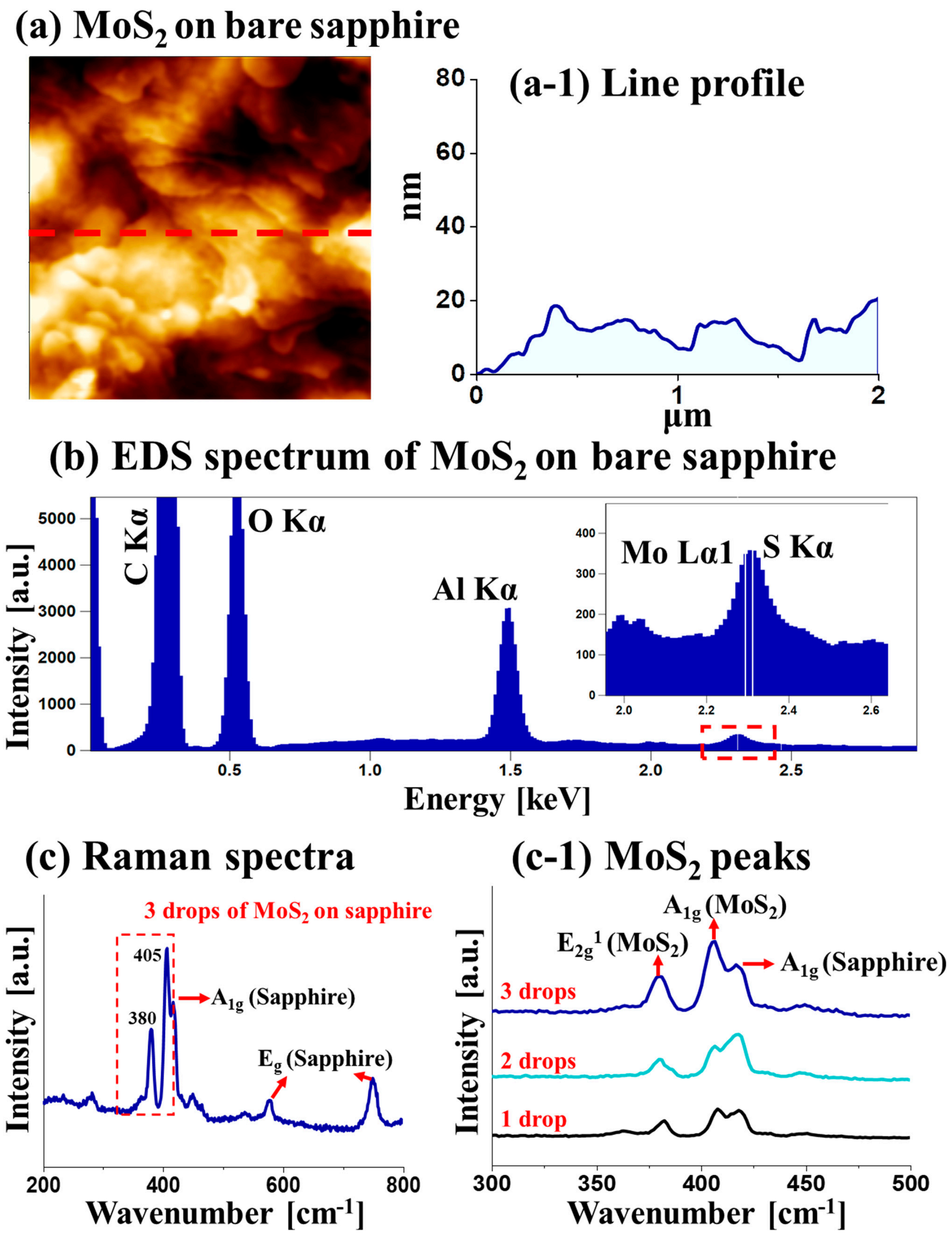


**Figure S17:** (a) AFM image of $MoS_2$ nanoplates deposition on bare sapphire. (a-1) Corresponding line-profile. (b) EDS spectrum of $MoS_2$ nanoplates on sapphire. (c) – (c-1) Raman spectra of $MoS_2$, where one drop corresponds to 20 µl of 0.25 mg/ml $MoS_2$ nanoplate solution.

# 2:1 mixture application at lower R6G concentrations

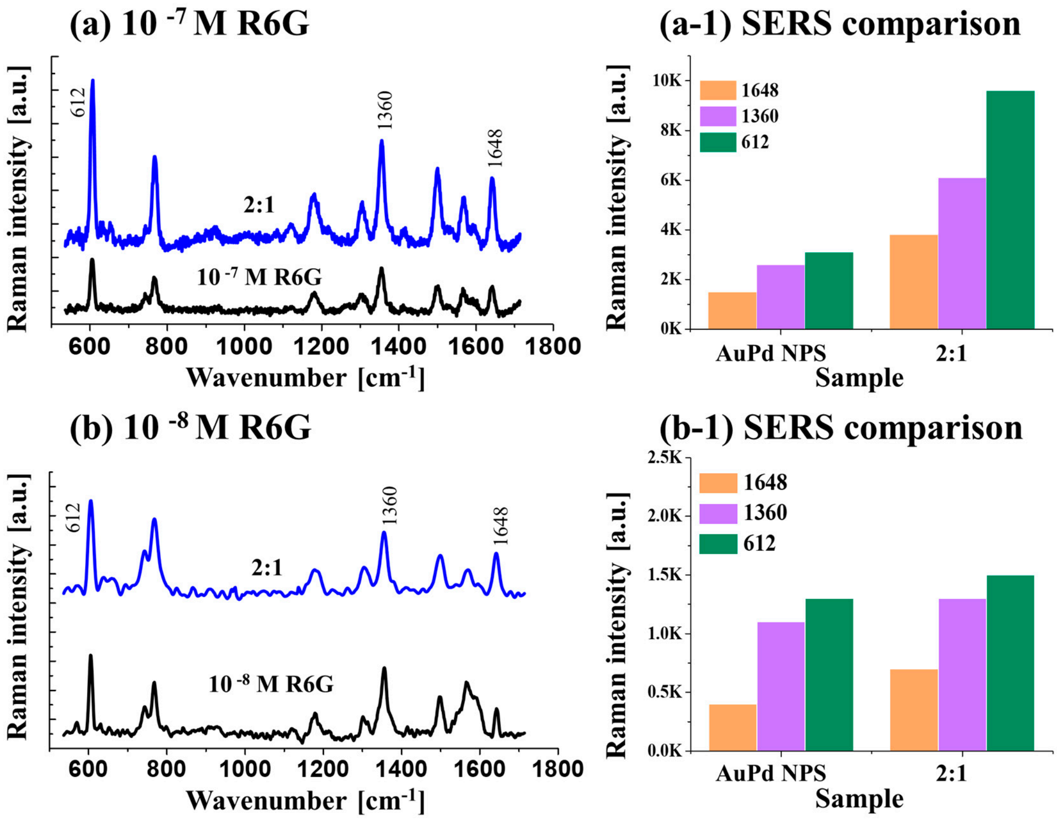


**Figure S18:** (a) – (b) SERS spectra of 2:1 mixture of $MoS_2$ nanoplatelets and R6G at $10^{-7}$ M and $10^{-8}$ M on HyCoS AuPd NPs. (a-1) – (b-1) Intensity counts of characteristic peaks.

# Raman of R6G on bare sapphire

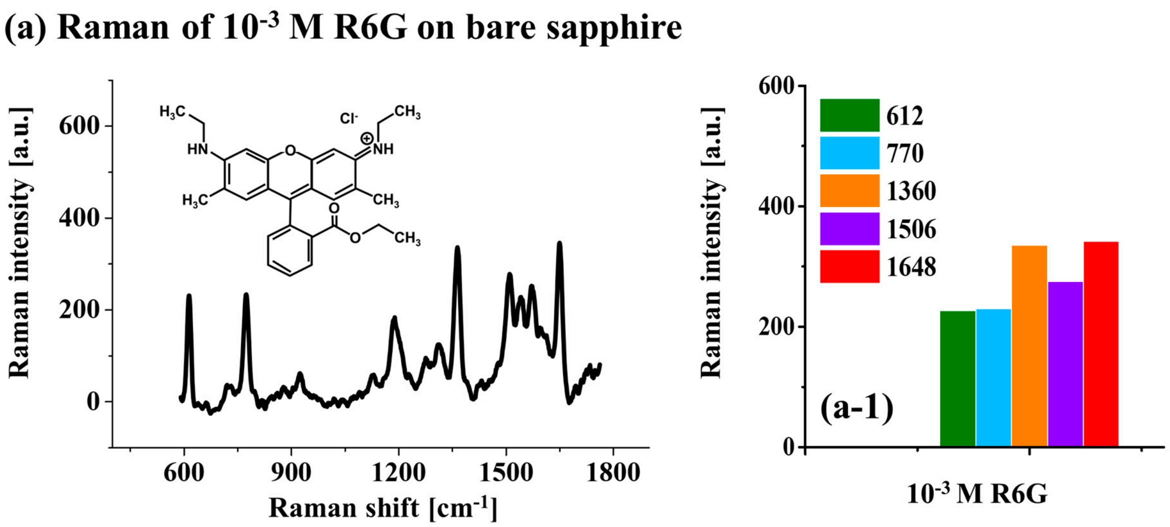


**Figure S19: (a)** Raman spectrum of R6G on bare sapphire. (a-1) Corresponding intensity plots.

# Limit of detection (LOD) calculation

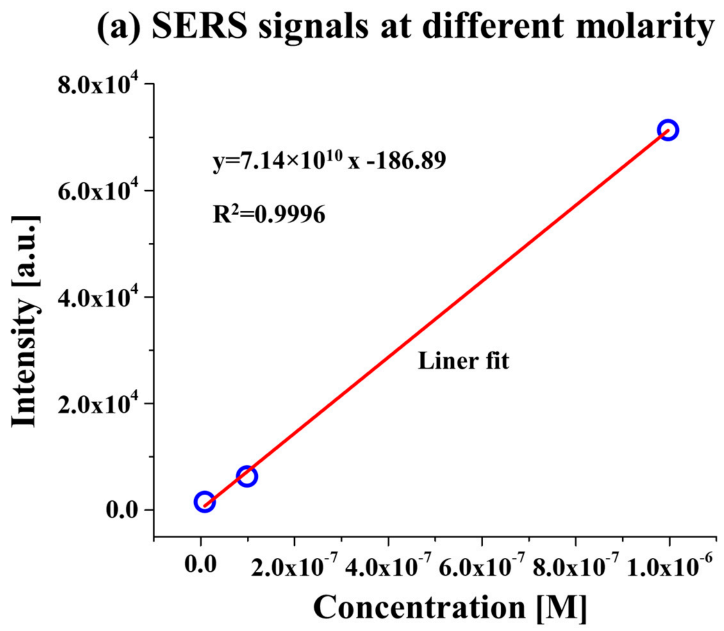


**Figure S20:** (a) Liner trend of the SERS signal at 1360 $cm^{-1}$ at concentration ranging from $10^{-8}$ to $10^{-6}$ M with the 2:1 mixture $MoS_2$ and $10^{-6}$ M R6G on AuPd HyCoS NPs**.**

**Table S1:** Summary table of SERS peak counts for $10^{-6}$ M R6G on various substrates. Counts are in thousand, i.e., $\times 10^3$. Related to Fig. 4(b).

| **Substrate** | **SERS peak position [$cm^{-1}$]** | | | | |
|---|---|---|---|---|---|
| | **612** | **770** | **1360** | **1506** | **1648** |
| **R6G on bare sapphire** | - | - | - | - | - |
| **R6G on $MoS_2$** | 1.933 | 1.629 | 2.148 | 1.612 | 2.96 |
| **R6G on HyCoS AuPd** | 9.8 | 5.9 | 9.227.2 | 5.0 | 6.1 |
| **$MoS_2$:R6G mixture (2:1) on HyCoS AuPd** | 99.516 | 58.647 | 72.264 | 60.523 | 68.211 |

**Table S2:** Enhancement factor (EF) of $10^{-6}$ M R6G on $MoS_2$, HyCoS AuPd and mixture solutions ($MoS_2$ : R6G ) on AuPd HyCoS NPs at different peak positions.

| **Sample** | **SERS peak position [cm $^{-1}$]** | | |
|---|---|---|---|
| | **612** | **1360** | **1648** |
| **R6G on $MoS_2$** | $3.08 \times 10^8$ | $2.32 \times 10^8$ | $3.12 \times 10^8$ |
| **R6G on HyCoS AuPd** | $1.56 \times 10^9$ | $7.79 \times 10^9$ | $6.43 \times 10^9$ |
| **$MoS_2$:R6G mixture (2:1) on HyCoS AuPd** | $1.59 \times 10^{10}$ | $7.82 \times 10^{10}$ | $7.19 \times 10^{10}$ |

**Table S3:** Summary table of SERS peak counts of R6G molarity variation on HyCoS AuPd NPs at different peak positions. Counts are in thousand, i.e., × $10^3$. Related to Fig. 4(d).

| **Substrate** | **SERS peak position [cm $^{-1}$]** | | | | |
|---|---|---|---|---|---|
| | **612** | **770** | **1360** | **1506** | **1648** |
| **$10^{-8}$** | 1.3 | 0.9 | 1.1 | 0.6 | 0.4 |
| **$10^{-7}$** | 3.1 | 2.1 | 2.6 | 1.5 | 1.5 |
| **$10^{-6}$** | 9.8 | 5.9 | 7.2 | 5.0 | 6.1 |
| **$10^{-5}$** | 46.4 | 26.8 | 41.6 | 28.5 | 34.5 |
| **$10^{-4}$** | 100.5 | 60.5 | 88.4 | 48.5 | 78.7 |

**Table S4:** Summary table of SERS peak counts of various mixture solutions ($MoS_2$ : $10^{-6}$ M R6G) on HyCoS AuPd NP substrate at different peak positions. Volume ratios for a total of 20 µl with 0.25 mg/ml $MoS_2$ nanoplatelet concentration. Counts are in thousand, i.e., × $10^3$. Related to Fig. 4(f).

| **Sample** | **SERS peak position [cm $^{-1}$]** | | |
|---|---|---|---|
| | **612** | **1360** | **1648** |
| **1:1** | 57.35 | 44.6 | 35.9 |
| **2:1** | 99.516 | 71.264 | 68.211 |
| **10:1** | 40.2 | 34.47 | 27.164 |

**Table S5:** Summary table of SERS peak counts of $10^{-7}$ and $10^{-8}$ M R6G with and without $MoS_2$ on HyCoS AuPd NPs. 2:1 ($MoS_2$ : R6G). Counts are in thousand, i.e., $\times 10^3$. Related to Fig. S17.

| Sample | SERS peak position [$cm^{-1}$] | | |
|---|---|---|---|
| | **612** | **1360** | **1648** |
| **$10^{-7}$ M** | 3.1 | 2.6 | 1.5 |
| **2:1** | 9.6 | 6.1 | 3.8 |
| **$10^{-8}$ M** | 1.3 | 1.1 | 0.4 |
| **2:1** | 1.5 | 1.3 | 0.7 |
